# Cooperation of DLC1 and CDK6 Affects Breast Cancer Clinical Outcome

**DOI:** 10.1534/g3.114.014894

**Published:** 2014-11-24

**Authors:** Xiaofeng Dai, Lu Li, Xiuxia Liu, Weiguo Hu, Yankun Yang, Zhonghu Bai

**Affiliations:** * National Engineering Laboratory for Cereal Fermentation Technology, Jiangnan University, Wuxi 214122, China; † School of Biotechnology, Jiangnan University, Wuxi 214122, China; ‡ Department of Obstetrics and Gynecology, University of Helsinki and Helsinki University Central Hospital, Helsinki, Finland

**Keywords:** breast cancer, survival, DLC1, CDK6, cooperation

## Abstract

Low DLC1 expression is found to frequently co-occur with aberrant expression of cell cycle genes including CDK6 in human lung and colon cancer. Here, we explore the influence of the synergistic effect of DLC1 and CDK6 on human breast cancer survival at the genetic, transcriptional, and translational levels. We found that high DLC1 and low CDK6 expression are associated with good prognosis. The DLC1 intronic SNP rs561681 is found to fit a recessive model, complying with the tumor suppressive role of DLC1. The heterozygote of the DLC1 SNP is found to increase the hazard when the CDK6 intronic SNP rs3731343 is rare homozygous, and it becomes protective when rs3731343 is common homozygous. We propose that DLC1 expression is the lowest in patients harboring the rare homozygote of rs561681 and functional DLC1 is the lowest when rs561681 is heterozygous and rs3731343 is rare homozygous. We are the first to report such synergistic effects of DLC1 and CDK6 on breast cancer survival at the transcriptional level, the overdominant model fitted by the SNP pair, and the dominant negative effect at the translational level. These findings link the germline genetic polymorphisms and synergistic effect of DLC1 and CDK6 with breast cancer progression, which provide the basis for experimentally elucidating the mechanisms driving differential tumor progression and avail in tailoring the clinical treatments for such patients based on their genetic susceptibility.

Studies of lung and colon cancer show that underexpression of DLC1 frequently co-occurs with deregulated expression of cell cycle genes such as CDK6, CDK4, CDKN2A, and CDKN2B. For example, poor prognosis was reported to be associated with low DLC1, low CDKN2A/B and high CDK4 in lung cancer, and low DLC1, low CDKN2B, and high CDK6 in colon cancer (Wu *et al.* 2009). DLC1, Deleted in Liver Cancer-1, is a tumor suppressor that is inactivated by genomic deletions or DNA methylation in many human malignancies, including liver, lung, breast, colorectal, and prostate cancer (Qian *et al.* 2012; Durkin *et al.* 2007; Kim *et al.* 2009; Seng *et al.* 2007). DLC1 catalyses the inactivation of RhoGTP to RhoGDP; however, it is less efficient in converting Cdc42GTP to Cdc42GDP (Qian *et al.* 2012). Rho family GTPases serve as molecular switches in various cellular functions, including cell cycle progression, cytoskeletal organization, malignant transformation, cell migration, and cell adhesion to the extracellular matrix (ECM) (Wu *et al.* 2009). Reactivation of DLC1 results in suppression of tumor cell proliferation and reduces tumorigenicity in liver and colon cancer (Wu *et al.* 2009; Zhou *et al.* 2004).

The cyclin-dependent kinases (CDKs) are important regulators of the mammalian cell cycle (Joyce *et al.* 2001). CDK4 and CDK6 form a holoenzyme complex with D cyclins (Pestell *et al.* 1999; Sherr 1996; Weinberg 1995) to phosphorylate pRB (Matsushime *et al.* 1991; Kiyokawa *et al.* 1992; Lukas *et al.* 1995), leading to the release of the heterodimeric transcription factor complex, E2F/DP, which contributes to the transcriptional control of genes that encode cell cycle regulatory proteins. CDK complexes are subject to regulation by Ink4 and p21 family proteins (Joyce *et al.* 2001). Although the p21 family proteins bind and inhibit the activity of cyclin-CDK complexes under a variety of stress stimuli (Labaer *et al.* 1997; Cheng *et al.* 1998), the Ink4 proteins dissociate the cyclinD1/CDK4/6 complex (Quelle *et al.* 1995; Quelle *et al.* 1997; Pomerantz *et al.* 1998). CDK4 and CDK6 share 71% amino acid identity (Grossel and Hinds 2006), are expressed ubiquitously (Meyerson and Harlow 1994; Meyerson *et al.* 1992), and function in the G1 phase of the cell cycle (Grossel and Hinds 2006); thus, they have historically been considered to function redundantly. Further investigations have revealed that certain types of tumors selectively amplify either CDK4 or CDK6 (Wolfel *et al.* 1995; Timmermann *et al.* 1997; Zuo *et al.* 1996; Easton *et al.* 1998), and they have distinct subcellular localization (Grossel *et al.* 1999; Ericson *et al.* 2003; Mahony *et al.* 1998; Fahraeus and Lane 1999). Also, CDK6 has been found to play a role in the differentiation of a variety of cell types where CDK4 does not (Grossel and Hinds 2006).

Here, we explore the functional relations between DLC1 and CDK6 in breast cancer survival at both germ line genetic and gene expression levels. Such interactions were also examined among their relevant proteins, such as those that directly bind to them at the translational level. The results help us in understanding the cooperation between DLC1 and CDK6 in breast cancer, which, once experimentally verified, contribute to personalized medicine and can be applied for clinical use.

## Materials and Methods

### Data

Supporting Information, Table S1 summarizes the major data sets used in this study, with details shown below.

### Genotype data

#### HEBCS data set:

The HEBCS (Helsinki Breast Cancer Study) data were collected in Helsinki, Finland, and are representative of breast cancer case series at the recruitment center during the collection periods (unselected cases collected 1997–1998 and 2000 as well as 2001–2004, with additional familial cases). All the breast cancer cases included have histopathological and survival data available; detailed information regarding the patient series and data collection is available elsewhere (Fagerholm *et al.* 2008). The average age at diagnosis is 56.8 years. Genotyping was conducted using the Illumina 550 platform as previously described (Fagerholm *et al.* 2008; Li *et al.* 2011). The intensity data generated were loaded into Illumina’s Genome studio and genotypes were generated with a GenCall threshold of 0.15. The data consist of 805 samples.

#### POSH data set:

The POSH (Prospective study of Outcomes in Sporadic *vs.* Hereditary breast cancer) data were collected from January 2000 to January 2008 from oncology clinics in the United Kingdom (Rafiq *et al.* 2013). The average age of the participants at diagnosis is 35.5 years.

Genotyping of 543 cases was conducted using the Illumina 660-Quad SNP array in two separate batches at two locations: 243 triple negative (ER, PR, and HER2 negative) patients were genotyped at the Mayo Clinic, Rochester, Minnesota (Haiman *et al.* 2011) and the rest of the patients were genotyped at the Genome Institute of Singapore. To ensure complete harmonization of the genotype calling, the intensity data available from all locations in form of *.idat files were further combined and used to generate genotypes based on the algorithm available in the genotyping module of Illumina’s Genome Studio software. A GenCall threshold of 0.15 and the HumanHap660 annotation file were used.

### Gene expression data

#### HEBCS data set:

The HEBCS gene expression data (GSE24450) were used for studying the synergistic effect between genes. There are 183 primary breast tumor samples, among which 151 were collected as a part of the unselected series at the Department of Oncology of the Helsinki University Central Hospital (HUCH) in 1997, 1998, and 2000 (Syrjakoski *et al.* 2000; Kilpivaara *et al.* 2005) and at the Department of Surgery from 2001 to 2004 (Fagerholm *et al.* 2008). The remaining 32 patients belong to an ongoing collection of additional familial breast cancer series from the department of Clinical Genetics at HUCH. Data for ER (Estrogen Receptor) status and for PR (Progesterone Receptor) status were collected from the pathology reports (Eerola *et al.* 2005).

Total RNA was extracted from the 183 primary breast tumors, and the samples were processed and hybridized to Illumina HumanHT-12_V3 Expression BeadChips containing 24,660 Entrez Gene entities according to the manufacturer recommendations (http://www.illumina.com). Gene expression profiling was performed at SCIBLU Genomics Centre, Lund University, Sweden.

Microarray raw data were imported into R (Team 2009) and processed by the methods included in the BioConductor facilities (Gentleman *et al.* 2004; Smyth 2005). Briefly, after quality control (Du *et al.* 2008), the data were normalized using the quantile method (Bolstad *et al.* 2003) and the gene expression matrix was obtained by averaging the probes mapped to the same Entrez Gene IDs (Maglott *et al.* 2005).

#### TCGA data set:

The level 3 primary solid breast tumor mRNA expression data were retrieved from TCGA (http://cancergenome.nih.gov) on 21 November 2011 and include 514 samples. The mRNA data were produced using the Agilent 244K Custom Gene Expression G4502A-07-3 platform, lowess-normalized, and log2 transformation of the ratio between two channels was performed.

#### Protein expression data:

The level 2 primary solid breast tumor reverse-phase protein microarrays (RPPA) data were retrieved from TCGA on 14 February 2013, and contain 385 samples. The Super curve log2 values were linearized, median-centered by the median across all samples, and normalized by the median across the entire panel of antibodies following the protocol (The University of Texas).

#### Copy number variation data:

Two types of breast cancer copy number variation (CNV) data, which were log2 transformed copy number values from Affymetrix SNP6 and putative copy-number calls determined using GISTIC 2.0 (−2, −1, 0, 1, 2 represent homozygous deletion, hemizygous deletion, neutral, gain, and overamplification, respectively) were used in this study. Both data sets included 889 samples from the TCGA provisional study and were retrieved via cBio (Cerami *et al.* 2012).

The discrete copy number calls with samples mapped to those having gene expression data were used as the covariate to adjust the CNV effect in the eQTL analysis. The continuous log2 transformed values were used for assessing the pair-wise correlations and examining the CNV among samples categorized by the genotype combination of the identified SNP pair.

### SNP retrieval

We first studied the interactions between DLC1 and CDK6 at the genetic level. SNPs within DLC1 and CDK6, including 10-kb expansion from both ends, were retrieved using SNPper (Riva and Kohane 2004). The tagging SNPs were obtained using SNAP (Proxy Search tool) (Johnson *et al.* 2008) where Caucasian samples (CEU, *i.e.*, northern and western Europe) were included from the 1000 Genomes Pilot 1, the linkage disequilibrium was restricted to r^2^ ≥0.8, and the distance from the nominal SNP was confined to 500 bp. The retrieved SNPs and their tagging SNPs were mapped to HEBCS and POSH genome-wide association study (GWAS) data, with SNPs present in both data sets selected for the interaction analysis.

### SNP interaction survival analysis

Two factors, age and BRCA status, were considered to be influential on patient survival, given the large differences between the average age of the two cohorts and the crucial roles played by BRCA1/2 and their potential influences on patient survival in breast cancer. We adjusted age (continuous variable) by taking it as a covariate and removed the confounding effect of BRCA status (binary variable) by taking it as a covariate and excluding BRCA carriers. Both factors were adjusted separately and in combination. Additionally, the basic Cox model without adjusting any factor was built as the reference. In the survival analysis, additive, dominant, recessive, overdominant (*i.e.*, separating the heterozygote from the homozygotes in the survival analysis) models were used to fit each SNP in the pair. The pooled analysis (POOL), *i.e.*, data from HEBCS and POSH were pooled together, was conducted to improve the statistical power in identifying the model that fits both datasets. Among all the possible combinations (*e.g.*, “dominant+additive” means fitting the DLC1 SNP to the dominant and the CDK6 SNP to the additive model), the model having the largest improvement in the significance in the pooled analysis as compared with using HEBCS or POSH data alone was selected. The interactions were performed between SNPs of DLC1 and CDK6 using Cox regression model in R (Team 2009).

SNP pairs having a significant (*P* ≤ 0.05) interaction term as compared with the model without it were first selected. Pairs with hazard ratios (HR) showing a consistent direction between HEBCS and POSH data sets and improved significance in the pooled analysis were chosen. For each selected interacting SNP pair, the SNP main effect was analyzed by fitting them, separately, to a Cox regression model. Analysis using multiple datasets and multiple criteria controls the false-positive rate of this study.

### Linkage disequilibrium checking among SNPs in the interacting pairs

Linkage disequilibrium (LD) was checked using SNAP (Pairwise LD tool) (Johnson *et al.* 2008) for SNPs within the selected pairs, and redundant pairs sharing the same haplotype (r^2^ >0.8) were removed. Default setting was used in SNAP as in the SNP retrieval stage.

### Expression quantitative trait loci analysis

The primary solid tumor genotype and level 3 gene expression data were retrieved from TCGA portal at http://tcga.cancer.gov/dataportal and used for the expression quantitative trait loci (eQTL) analysis. TCGA CNV data were retrieved using the cBio cancer genomics portal (http://www.cbioportal.org/public-portal/) (Cerami *et al.* 2012) and was used as the covariate; 502 samples shared the genotype, gene expression, and CNV data, which were used in the analysis. The genotype data contain 906,600 SNPs, where genotypes with confidence score more than 0.1 were considered as missing data (Xu *et al.* 2009; Affymetrix 2008). The eQTL analysis was performed with and without CNV as the covariate.

### Expression interaction survival analysis

Gene expression interaction survival analysis was performed between DLC1 and CDK6 using TCGA data. The gene expression data were partitioned into binary values (high and low expression) by an optimized percentile and then fitted into the Cox regression model including the effect of each gene and their interactions. As a comparison, the model without the interaction term was built. The significance of the interaction was assessed by the Chi-square test of the likelihood ratio between the model with and without the interaction term. The Kaplan-Meier plots on DLC1 expression for tumors overexpressing and underexpressing CDK6 and those regardless of CDK6 expression were drawn for each pair.

Protein expression interaction survival analysis was performed between proteins that directly bind or influence DLC1 (DLC1 binds Caveolin 1) (Du *et al.* 2012), CDK6 (CDK6 binds CDKN1B) (Soos *et al.* 1996), and Cyclin D1 (Weinberg 2007) using TCGA RPPA data.

### Correlation analysis

Correlation was analyzed at gene expression, protein, and CNV levels. The spearman correlation scores between each pair were computed and a linear model was built for each pair as well. The cutoff was set to *P* ≤ 0.05 to assess the significance of the correlation.

#### Expression analysis:

The gene expression, protein expression, and CNV profiles were checked for the genotype combinations of the identified SNP pair. Data at each level were grouped according to the genotype combination of the SNP pair, and the group-wise pattern was visualized via boxplot. The significance of the heterogeneity across the distribution of the expression among groups was evaluated by the Kruskal-Wallis rank sum test, with the null hypothesis being that the location parameters of the distribution is the same in each group. The significance level was set to *P* ≤ 0.05 for all the data sets.

#### Functional tagging SNP exploration:

FASTSNP (Yuan *et al.* 2006) was used to study the functional roles of the identified SNPs or their tagging SNPs. The tagging SNPs being analyzed were retrieved using SNAP (Proxy Search tool) (Johnson *et al.* 2008) with r^2^ >0.8 or d′ >0.8. SNPs located in the coding region and result in missense mutations or that resided in the intron and cause the loss or addition of human transcription factors were selected.

## Results

### Interactions between DLC1 and CDK6

#### Analysis at the genetic level:

We retrieved the SNPs and their tagging SNPs for DLC1 and CDK6 with r^2^ >0.8 as described in the *Materials and Methods* section. There are 28 DLC1 SNPs and 26 CDK6 SNPs, respectively, mapped to both the HEBCS and POSH data. A pair-wise analysis was performed between DLC1 and CDK6 SNPs, resulting in 728 pairs in total. The synergistic effect was found in 14 pairs using all samples, 10 pairs in ER-negative tumors, and 6 pairs in ER-positive tumors (the cut-off *P* value is 0.05; detailed information on SNP pair selection is presented in the *Materials and Methods* section) as listed in Table S2. Among them, only one pair, rs561681 (DLC1) and rs3731343 (CDK6), shows significant associations between each SNP and the corresponding gene. As shown by the “additive+additive” models in Table 1, the data fit significantly better to the model having the interaction term as compared with the one that does not, especially when data from HEBCS and POSH are pooled together (*P* = 4.96E-11). Also, the M1 model (model including the interaction term) improves when fitting the DLC1 SNP to the overdominant model and the CDK6 SNP to the additive model (“overdominant+additive”) rather than fitting them both to the additive model (“additive+additive”). The detailed statistics of the selected model (“overdominant+additive” of M1) were shown in Table S3, where the common and rare alleles of the DLC1 SNP were denoted as “A” and “a” and those for the CDK6 SNP were represented as “B” and “b”, respectively. It is seen that the combination of the heterozygote of rs561681 (DLC1) and rare homozygote of rs3731343 (CDK6) is associated with poorer clinical outcome (Table S3).

**Table 1:** Model selection

Data Type	SNP												
M1+M2	overdominant+additive			recessive+additive			dominant+additive			additive+additive			GEX
**Data**	HEBCS	POSH	POOL	HEBCS	POSH	POOL	HEBCS	POSH	POOL	HEBCS	POSH	POOL	TCGA
***P* (M1)**	0.02	0.205	4.93E−12	0.13	0.642	7.02E−10	0.561	0.34	1.42E−09	0.0489	0.271	4.96E−11	0.0024
***P* (M2)**	0.422	0.698	7.57E−10	0.201	0.604	2.34E−10	0.895	0.956	3.35E−09	0.301	0.718	7.50E−10	0.9178
***P* (M1M2)**	0.0051	0.0556	2.95E−04	0.1436	0.4659	0.2015	0.1907	0.069	0.0269	0.0301	0.0983	0.0029	6.15E-05
M1:G1+G2+G1*G2													
M2:G1+G2													

In “Data type,” “SNP” and “GEX” each represent the SNP and gene expression data, respectively. In “M1+M2,” the name of the genetic model for each SNP is shown. *P* (M1) and *P* (M2) are the *P* values of the models M1 and M2, respectively, from the likelihood ratio test, and *P* (M1M2) shows the *P* value of the Chi-square test by comparing M1 (the model with the interacting term) and M2 (the model without the interacting term). The “additive+additive” model is the reference null model, and the “overdominant+additive” model is the selected model.

“SNP” stands for the genotype and phenotype data, “GEX” represents gene expression data, “PEX” is short for protein expression data, and “CNV” means copy number variation. The total number of samples is shown for each data set, and the number of events in data used for survival analysis is shown in parentheses.

**Figure 1:**
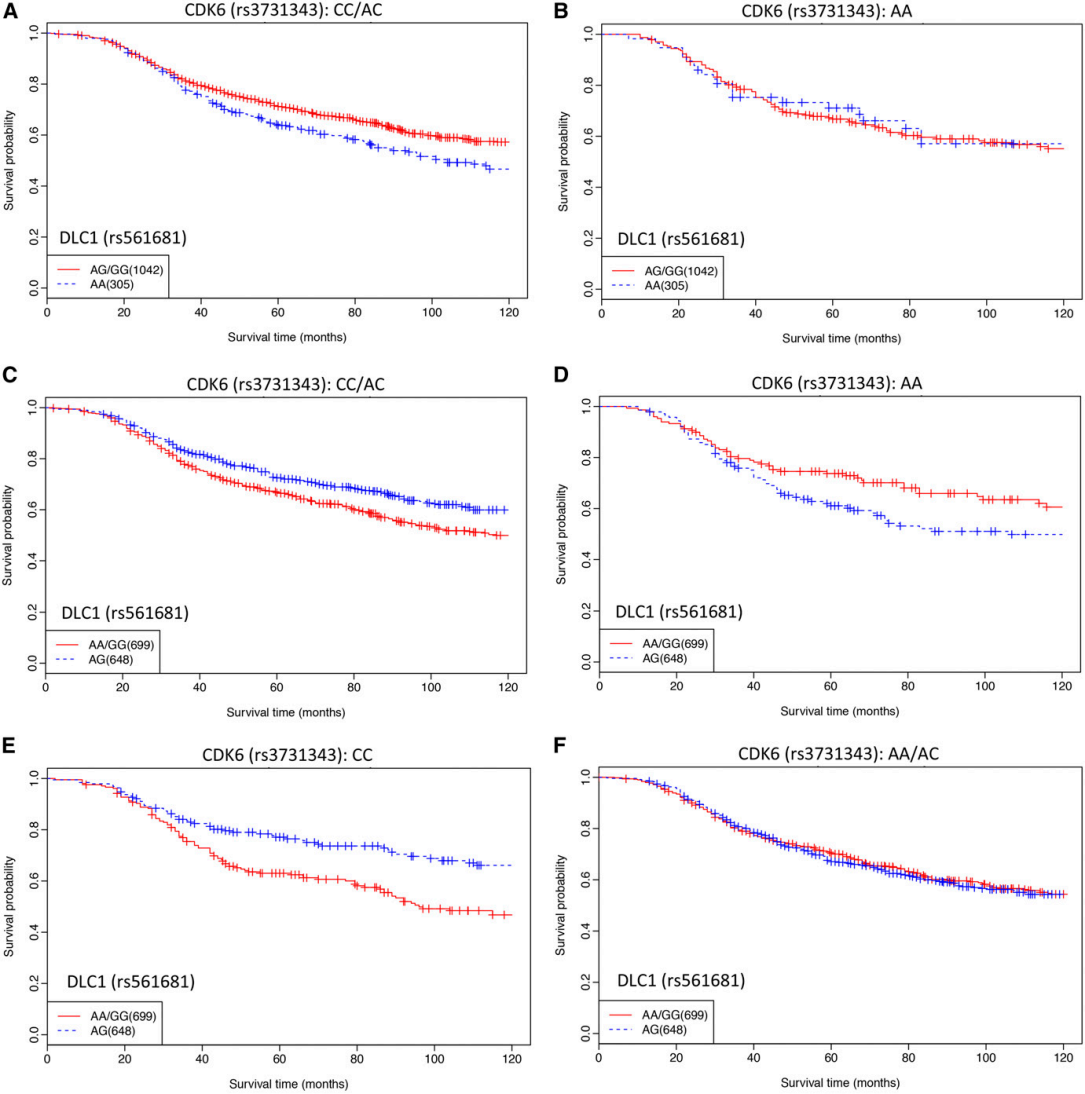
Kaplan Meier plots of patients’ survival showing interactions in the identified SNP pair between DLC1 and CDK6. The plots were drawn for the DLC1 SNP (rs561681) stratified by the genotypes of the CDK6 SNP (rs3731343). Results from HEBCS and POSH data were pooled. Plots showing (A–B) the interactive effect of AA:AC/CC (aa:bB/BB, the rare homozygote of the DLC1 SNP *vs.* the combination of the heterozygote and common homozygote of the CDK6 SNP), (C–D) the interactive effect of AG:AA (aA:bb, the heterozygote of the DLC1 SNP *vs.* the rare homozygote of the CDK6 SNP), and (E–F) the interactive effect of AG:CC (aA:BB, the heterozygote of the DLC1 SNP *vs.* the common homozygote of the CDK6 SNP).

To explore further the significance of the interactions of various genotype combinations on patient survival, the Kaplan Meier plots of the DLC1 SNP as stratified by the genotypes of the CDK6 SNP were drawn (Figure 1), with the statistics summarized in Table 2. It is shown that the heterozygote of the DLC1 SNP has an amplifying effect on the CDK6 SNP, *i.e.*, it increases the hazard when combined with the rare homozygote of the CDK6 SNP (see Figure 1D and compare it with Figure 1C, Table 2) and becomes protective when interacting with the common homozygote of the CDK6 SNP (see Figure 1E and compare it with Figure 1F, Table 2). Significant protective effect was also observed when fitting both SNPs in the pair to the recessive model, *i.e.*, neither SNP is rare homozygous (see Figure 1A and compare it with Figure 1B, Table 2). As represented by the stratification analysis (Table 2), this effect, although marginally significant in POSH data (*P* = 0.097, HR = 0.75, 95% C.I.= 0.53, 1.06), has similar patterns in both data sets (Figure S1) and the significance is largely improved in the pooled analysis (*P* = 0.0035, HR = 0.73, 95% C.I. = 0.59, 0.9). This interaction is significant with and without BRCA patients being removed, indicating its independence of BRCA status. Also, whether the confounding effect of age is adjusted does not make a significant difference here. Importantly, although being less significant than interacting with the nonrare homozygote of the CDK6 SNP, the rare homozygote of the DLC1 SNP significantly increases hazard in HEBCS data and the pooled analysis (Table 2).

**Table 2:** Statistics of the SNP pair (rs561681 and rs3731343) representing interactions between DLC1 and CDK6

Genotype	HEBCS			POSH			POOL			Meta	
Symbol	*P*	HR	95% C.I.	*P*	HR	95% C.I.	*P*	HR	95% C.I.	*P*_HR	*P*_size
AG/GG:CC/AC	0.019	0.72	0.55, 0.95	0.097	0.75	0.53, 1.06	0.0035	0.73	0.59, 0.90	0.0084	0.0076
aA/AA:BB/bB											
AG:AA	0.011	1.39	1.08, 1.79	0.059	1.34	0.99, 1.82	0.0016	1.35	1.24, 1.66	0.0032	0.0029
aA:bb											
AG:CC	0.004	0.53	0.34, 0.81	0.03	0.57	0.35, 0.95	0.0004	0.56	0.39, 0.76	0.0007	0.0006
aA:BB											
AA	0.04	1.3	1.01, 1.67	0.22	1.21	0.89, 1.65	0.016	1.27	1.05, 1.54	0.0362	0.0327
aa											
AG	0.13	0.84	0.67, 1.05	0.3	0.87	0.67, 1.13	0.076	0.86	0.72, 1.02	0.1223	0.1144
aA											
AA	0.86	1.03	0.78, 1.36	0.88	0.98	0.71, 1.33	0.97	1	0.82, 1.24	0.9445	0.943
bb											
CC	0.46	0.91	0.71, 1.17	0.57	1.09	0.82, 1.46	0.91	0.99	0.82, 1.19	0.5248	0.5137
BB											
Genotype (Gene): Symbol				G (DLC1): A			A (DLC1): a				
				C (CDK6): B			A (CDK6): b				

The true genotypes are shown under “Genotype,” and the symbols “A” and “a” represent the common and rare allele in the DLC1 SNP (rs7654599), and “B” and “b” stand for the common and rare allele in the CDK6 SNP (rs17213431), respectively, under “Symbol.” *P*, HR, and 95% C.I. are the *P* value, hazard ratio, and 95% confidence interval (low, high) for each analysis.

**Figure 2:**
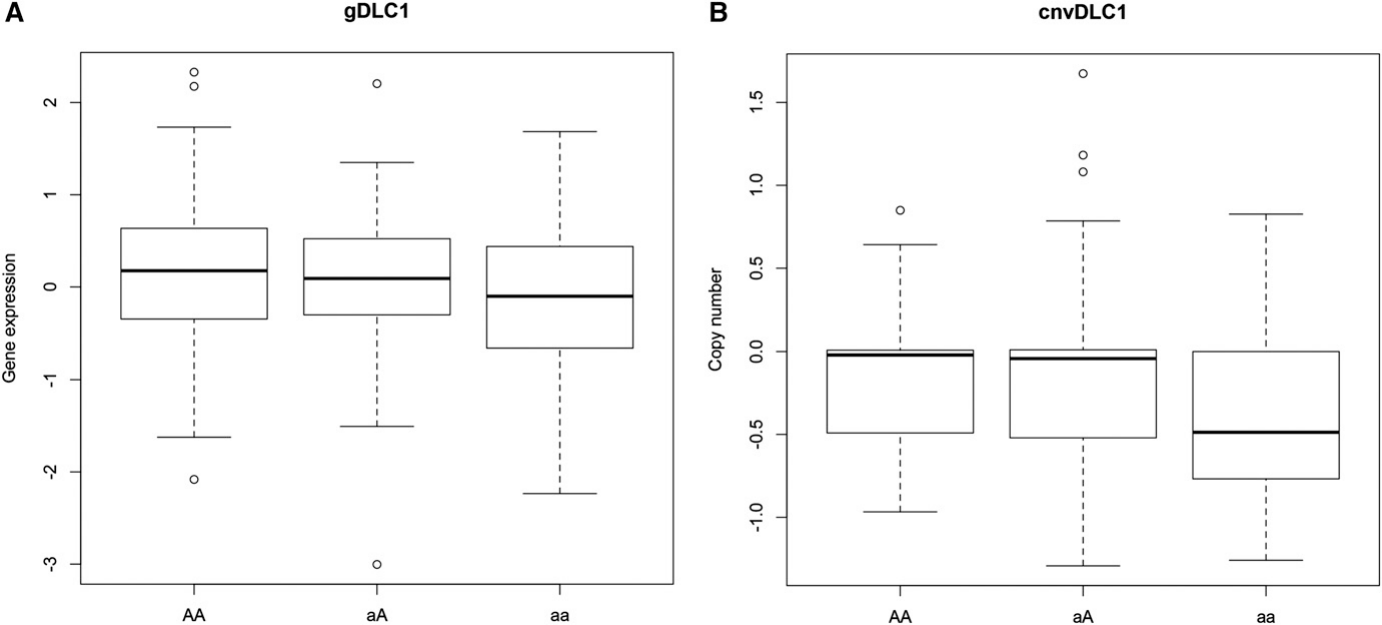
Gene expression pattern and copy number variation of DLC1 categorized by the genotypes of the DLC1 SNP (rs561681). (A) Gene expression pattern categorized by the genotypes of rs561681. (B) Copy number variation categorized by the genotypes of rs561681. The vertical axis of (A) shows the gene expression value, which is lowess-normalized, followed by log2 transformation of the ratio between two channels, and that of (B) shows the log2 transformed copy number values from Affymetrix SNP6. Both datasets are retrieved from TCGA.

The distribution of DLC1 expression significantly differs by the allele distribution of its intronic SNP rs561681 (the *P* value from the Kruskal-Wallis rank sum test is 0.023), where DLC1 expression increases with the dose of the common allele of rs561681 (Figure 2A). Similarly, the distribution of the copy number of DLC1 distinctively varies among patients having different allele combinations of rs561681 (with a *P* value from the Kruskal-Wallis rank sum test being 0.00014), and the copy number is most frequently deleted in patients harboring the rare homozygote of the DLC1 SNP as compared with the others (Figure 2B). Further, the copy number of DLC1 is significantly positively correlated with that of CDK6 (*P* = 0.0008, correlation coefficient is 0.18; Table S4).

We checked the allele frequencies of the identified SNP pair in the 1000 Genome Project through HapMap (release 27). The allele frequencies of both alleles in rs561681 are similar, *i.e.*, 41.2% for “a” and 58.8% for “A,” in CEU (*i.e.*, Utah residents with Northern and Western European ancestry from the CEPH collection) according to the 1000 Genome Project (1000 Genomes Project Consortium *et al.* 2012). For some populations, they can be 50% for each (GBR, *i.e.*, England and Scotland) or in an opposite distribution, *e.g.*, 57.1% for “a” and 42.9% for “A” in CHB (*i.e.*, Chinese in Beijing) (1000 Genomes Project Consortium *et al.* 2012). The genotype frequencies of AG (aA) and AA (bb) are 0.54 and 0.195, respectively, in CEU (1000 Genomes Project Consortium *et al.* 2012), leading to a frequency of 10.53% (0.54×0.195) for aA:bb in this population. We also checked these statistics using our data sets (*i.e.*, HEBCS and POSH, which belong to the same ethnic group with CEU in the 1000 Genome Project). As expected, the allele frequencies of rs561681 are 48.35% (“a”) and 51.58% (“A”) in HEBCS, and 46.42% (“a”) and 54.95% (“A”) in POSH. The combined genotype aA:bb accounts for 10.32% and 10.87%, respectively, in the HEBCS and POSH data sets (Table 3).

**Table 3:** Genotype distribution of the SNPs and their genotype combinations in HEBCS and POSH data sets

	HEBCS		POSH	
	n	%	n	%
**DLC1 (rs561681)**				
**GG (AA)**	230	28.61	164	30.20
**AG (aA)**	386	48.01	262	48.25
**AA (aa)**	188	23.38	117	21.55
**CDK6 (rs3731343)**				
**CC (BB)**	248	30.85	150	27.62
**AC (bB)**	397	49.38	261	48.07
**AA (bb)**	159	19.78	132	24.31
**rs561681:rs3731343**				
**GG:CC (AA:BB)**	69	8.58	46	8.47
**AG:CC (aA:BB)**	115	14.30	76	14.00
**AA:CC (aa:BB)**	64	7.96	28	5.16
**GG:AC (AA:bB)**	111	13.81	76	14.00
**AG:AC (aA:bB)**	188	23.38	127	23.39
**AA:AC (aa:bB)**	98	12.19	58	10.68
**GG:AA (AA:bb)**	50	6.22	42	7.73
**AG:AA (aA:bb)**	83	10.32	59	10.87
**AA:AA (aa:bb)**	26	3.23	31	5.71

#### Analysis at the transcriptional level:

Significant interactions were also observed between DLC1 and CDK6 at the transcriptional level. As shown in Table 1, the model improves (*P* = 6.15E−5) and becomes significant (*P* = 0.00241) when including the interaction term in the Cox regression model. Poor survival corresponds to low DLC1 and CDK6 expression (Table 4).

**Table 4:** Statistics of the selected model including the interactions between DLC1 and CDK6 at the transcriptional level

Gene	Level	HR	95% C.I.
DLC1	Low (<74%)	0.18	0.29, 0.92
CDK6	Low (<50%)	0.36	0.09, 0.72
DLC1:CDK6	low:low	10.93	3.06, 38.98

Gene shows the gene and gene pair. Level shows the expression level, with the expression percentile shown in parentheses. HR and 95% C.I. are the hazard ratio and 95% confidence interval (low, high) for each gene or gene pair, respectively.

**Figure 3:**
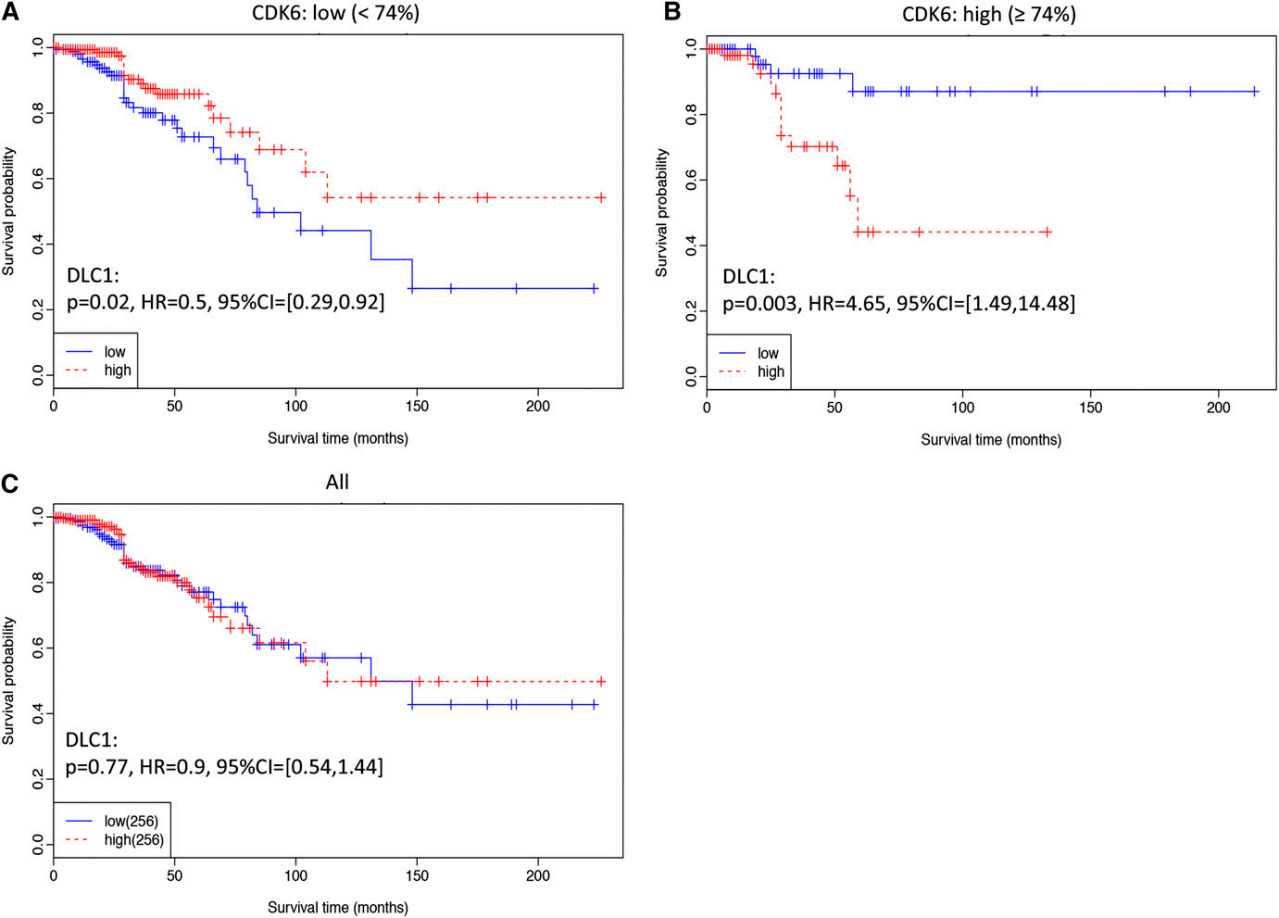
Kaplan Meier plots of patients’ survival showing interactions between DLC1 and CDK6 gene expression. (A) Kaplan Meier plots on patient survival for DLC1 gene expression when the expression level of CDK6 is less than the 74% percentile of all the samples. (B) Kaplan Meier plots of patient survival for DLC1 gene expression when the expression level of CDK6 is no less than 74% percentile of all the samples. (C) Kaplan Meier plots of patient survival for DLC1 gene expression when all samples are included. Median was used to split the gene expression of CDK6 into high and low expressions in both subplots.

The Kaplan-Meier plots on patient survival regarding DLC1 expression when CDK6 level is low or high and the plot regardless of CDK6 expression are compared and shown in Figure 3. High DLC1 expression corresponds to better prognosis when CDK6 is not particularly high (<74% percentile of all expression, *P* = 0.003; Figure 3A), worse survival when CDK6 is high (≥74% percentile of all expression, *P* = 0.02; Figure 3B), and no survival difference when all samples were included (*P* = 0.77; Figure 3C). Similar interactive effects were also observed in patient survival regarding CDK6 expression as stratified by the level of DLC1 (Figure S2).

We next examined the correlations between DLC1 and CDK6 expression. DLC1 was not significantly correlated with CDK6 expression when all tumor types were considered (*P* = 0.0817, correlation = 0.0769) but was significantly positively correlated with CDK6 in ER-positive tumors (*P* = 2.18E−9, correlation = 0.2967), indicating the involvement of ER in this interaction.

#### Analysis of the relevant genes at the translational level:

Restricted by the protein data available for DLC1 and CDK6, we analyzed the interactive effects of some proteins directly bind to DLC1 or CDK6. Caveolin 1 binds DLC1, and cyclin D1 and CDKN1B bind CDK6 (Du *et al.* 2012; Soos *et al.* 1996; Weinberg 2007). Significant interactions were observed between caveolin 1 and CDKN1B, as well as between caveolin 1 and cyclin D1, because the fitness between the data and model largely improves when the interaction term is included in the model (Table S5). The statistics of the model including the interaction term are shown in Table S6. The interactive effect is visualized by the Kaplan-Meier plots on patient survival using the expression of one protein as stratified by the others (Figure S3). Increased hazard was observed for patients with high caveolin 1 and low CDKN1B expression (Figure S3, A and B). Similarly, significant favorable prognosis is associated with patients expressing low cyclin D1 and high caveolin 1 (Figure S3, C and D). None of the three proteins affects patient survival on its own (Figure S4).

Among all the proteins with RPPA data available in TCGA, 10 proteins, including CDKN1B, PECAM1, p53, Claudin7, CHK1, Transglutaminase 2, ACC, PRDX1, GSK3, and PKCα, show distinct protein expression for patients harboring the “aA.bb” genotype combination as compared with the other genotype combinations of the SNP pair (Table S7, Figure S5). Additionally, caveolin1, experimentally verified to bind with DLC1 (Figure S6) (Du *et al.* 2012), has higher expression in aA:bb tumors as compared with the others (Figure S5D).

We studied the correlations among proteins directly related to DLC1 and CDK6 (Figure S6) by the existing experimental data collected via IPA (Ingenuity Systems, www.ingenuity.com). As expected, these proteins are significantly correlated with each other (Table S8), and all the input proteins are involved in cellular development, cellular proliferation, and cell cycle (score 17) according to IPA.

## Discussion

### DLC1 interacts with CDK6 in breast cancer

An interaction between DLC1 and CDK6 was found to affect breast cancer survival. Accordingly, one SNP pair, rs561681 (DLC1) and rs3731343 (CDK6), was identified whose nonrare homozygotes combination (aA/AA:bB/BB) is protective (Table 2). This implies that both SNPs fit the recessive model and patients without concurrent mutations in DLC1 and CDK6 may have favorable outcome. This is confirmed at the transcriptional level because patients without CDK6 overexpression and having excessive DLC1 expression tend to have better outcome than others (Figure 3A). Further, the allele distribution of the DLC1 SNP (rs561681) highly correlates with DLC1 expression (*P* = 0.004; Table S9). Particularly, the expression increases with the dose of the common allele (Figure 2A), consistent with the tumor suppressive role of DLC1. Low copy number of DLC1 is significantly (*P* = 0.0001) associated with the rare allele of DLC1 (Figure 2B, Table S9). This is in agreement with the fact that poorer outcome is associated with the rare allele of DLC1, and DLC1 is frequently lost in cancer (Qian *et al.* 2012; Durkin *et al.* 2007; Kim *et al.* 2009; Seng *et al.* 2007). Taken together, the protective effect of the common allele of the DLC1 SNP is amplified by the common allele of the CDK6 SNP. The identified SNPs both fit the recessive model, and the association of the rare allele of rs561681 (DLC1) with poorer clinical outcome is related to or driven by copy number deletion.

At the protein level, we examined the interactions between proteins directly related to DLC1 or CDK6. These include caveolin 1 [which directly binds DLC1 (Figure S5) and contributes to its tumor suppressive roles (Du *et al.* 2012)], CDKN1B [which is an inhibitor of and directly binds to CDK6 (Soos *et al.* 1996)], and cyclin D1 [which forms a complex with CDK6 (Weinberg 2007)]. Interestingly, the proteins related to CDK6 significantly interact with that of DLC1 at the translational level (Figure S3), additionally supporting the observed interactions between DLC1 and CDK6 and implying complex regulatory relationships among these proteins.

### DLC1 interacts with CDK6 via a dominant-negative effect

In the identified SNP pair, rs561681 (DLC1) fits an overdominant model in the interactions, *i.e.*, aA:BB is protective and aA:bb is risky. The heterozygote of the DLC1 SNP exhibits an amplifying effect on the CDK6 SNP, suggesting a dominant-negative effect of their interactions at the translational or phenotypic level.

To confirm this hypothesis, we examined the expression of some related proteins with available data in TCGA. The protein profiles categorized by the genotype combinations of the identified SNP pair show distinct patterns for patients having the aA:bb genotype (Figure S5, Table S7), which are in accordance with their functional roles and relationships with DLC1 and CDK6, additionally supporting the dominant-negative interactive effect between DLC1 and CDK6. For example, CDKN1B directly binds and inhibits CDK6 (known to have a tumor suppressive role) (Lin *et al.* 2001; Soos *et al.* 1996), whose underexpression implies a poor prognosis. This is consistent with the increased hazard observed from patients harboring the aA:bb (DLC1:CDK6) genotype. CDKN1B is regulated by p53 (Abraham *et al.* 2005), an important tumor suppressor regulating cell cycle and the guardian of the genome (Weinberg 2007) that is underexpressed in aA:bb (DLC1:CDK6) patients. We also checked the genotype distribution of the DLC1 SNP (rs561681) regarding some important histopathological markers (Table S11), among which the amount of cyclin D1, a binding partner of CDK6, distinctly varies by the genotypes of rs561681 (*P* = 0.002) without linear associations with the allele dose (*P* = 0.415). This suggests a distinct distribution of cyclin D1 for aA tumors at the protein level, further supporting our hypothesis.

Dominant-negative effect has been previously discovered in many genes and diseases such as Keap1 in lung cancer (Suzuki *et al.* 2011) and TNFRSF13B in CVID (common variable immunodeficiency) (Garibyan *et al.* 2007). The interaction of DLC1 with its substrates depends on its dimerization and is modulated by the switch from dimer to monomer mediated by Ser88 phosphorylation (Song *et al.* 2008). The amino-terminal domain of DLC1 (1-638) is known to have a dominant-negative effect in blocking cell migration by antagonizing endogenous DLC1 (Kim *et al.* 2008). Thus, it is possible that the variant could produce a dominant-negative mutant that efficiently binds wild-type DLC1 monomers to form heterodimers that are deficient in its own function but amplifies the effect of CDK6, *i.e.*, protective when CDK6 functions normally and risky when CDK6 is mutated.

The balanced allele distribution of the DLC1 SNP rs561681, as demonstrated by the statistics from both the 1000 Genome Project and our data sets, provides the basis for the existence of dominant-negative mutant in DLC1. However, the dominant-negative effect revealed using CEU data (our data belong to the CEU ethnic group) is not driven by the allele distribution, because the combined genotype aA:bb only accounts for approximately 10% of patients in this population (both 1000 Genome Project and our datasets) and ranked in the middle among all the combinations regarding the sample size (Table 3).

The SNP rs561681 (C_000008.11:g.13093516 by the HGVS name) is located in the 4^th^ intron of DLC1. One of its tagging SNPs, rs532841 (r^2^ = 0.652, d′ = 0.955; Table S10), is a missense mutation causing the amino acid change from valine (V) to methoionine (M) at position 791 (V791M), and the exonic splicing enhancer or silencer (ESE/ESS) motif is predicted to be changed using FASTSNP (Yuan *et al.* 2006). Another mutation of R718E was reported to result in a dominant-negative mutant in DLC1 due to the loss of the catalytic function in the RhoGAP domain (609–878) and the retain of the tensin binding site (Tyr^442^) (Kim *et al.* 2008). Because V791M is closely located around R718E, it is possible that a dominant-negative mutant is generated from the same mechanism here. However, another tagging SNP rs621554 (r^2^ = 0.817, d′=1; Table S10) causes the loss of the binding sites of transcription factors AP1 and CREB according to FASTSNP (Yuan *et al.* 2006). Besides rs621554, 91 tagging SNPs are located in the human transcription factor binding sites in the intron of DLC1 (Table S12). Apart from AP1 (Tu *et al.* 2009; Lu *et al.* 2002; van der Burg *et al.* 1995) and CREB (Conkright and Montminy 2005; Pradeep *et al.* 2004; Ionov *et al.* 2004; Jean and Bar-Eli 2001), the most frequently varied binding sites also include GATA1, GATA2, and GATA3, which are essential in cell-cycle control whose alteration contributes to the tumorigenesis process (Friedman 2010; Rylski *et al.* 2003; Kumar *et al.* 2012; Wang *et al.* 2012; Jacquemier *et al.* 2009; Ciocca *et al.* 2009; Abba *et al.* 2006; Mehra *et al.* 2005). These reports are suggestive and supportive of our hypothesis that the rare allele of rs561681 could reduce DLC1 expression by changing the binding sites of transcription factors such as AP1 and CREB, and the heterodimer produced by the heterozygote (aA) amplifies the effect of CDK6. However, the exact mechanism needs further in-depth exploration.

### Interactions between DLC1 and cell-cycle genes are influenced by ER status in breast cancer

The gene expression of DLC1 is positively influenced by that of CDK6 in ER-positive breast tumors (Table S13), indicating the importance of ER in the interactions between DLC1 and CDK6 in breast cancer. It is reported that DLC1 directly interacts with liganded ER and facilitates estrogen-induced ER transactivation and anchorage-independent cell growth (Rayala *et al.* 2005); also, DLC1 expression leads to enhanced recruitment of DLC1-ER complex to the ER-target gene chromatin, and estrogen induces the transcription and expression of DLC1 (Rayala *et al.* 2005). Some genes interacting with DLC1 or CDK6 are known to cooperate with ER as well. For example, cyclin D [which forms a complex with CDK4/6 (Weinberg 2007)] was suggested to operate upstream of ER (Neuman *et al.* 1997) and to enhance transactivation through an estrogen response element in a CDK-independent manner (Zwijsen *et al.* 1997; Neuman *et al.* 1997), whose overexpression was shown to correlate with an ER-positive status in breast cancer (Fantl *et al.* 1990; Courjal *et al.* 1996; Buckley *et al.* 1993). Finally, Figure S5 shows the current experimentally verified connections among the genes with interest from various tissues or cell lines collected in IPA. Given the heterogeneity of breast cancer and the important roles of ER in breast tumor subtype classification (Blows *et al.* 2010), ER may have a more intimate connection with these genes in breast cancer. We believe the involvement of ER in the synergistic effect between DLC1 and CDK6 could be revealed given datasets of larger sample size and more ethnic groups.

## Conclusion

The interactions between DLC1 and CDK6 were detected and explored at the genetic level and confirmed at the transcriptional level. Interactions of relevant proteins at the translational level provide additional evidence supporting the interactions between DLC1 and CDK6 and imply a complex regulatory relationship among them. In particular, one SNP pair, rs561681 (DLC1) and rs3731343 (CDK6), was identified to have a synergistic effect on breast cancer patient survival, as verified in two independent cohorts, and these SNPs were each associated with the expression of the corresponding gene. The interaction between the nonmutant forms of the two SNPs is protective. The DLC1 SNP (rs561681) fits a recessive model, complying with the tumor suppressive role of DLC1. Tumors that are rare homozygous for the DLC1 SNP (rs561681) exhibit lower gene expression and more frequent copy number loss of DLC1. Significant survival differences were observed on DLC1 expression as stratified by that of CDK6, with more favorable prognosis for patients without CDK6 overexpression and high DLC1 level. In addition, the protein that directly binds to DLC1 (*i.e.*, caveolin 1) was shown to have significant interactive effects with those related to CDK6 (*i.e.*, cyclin D1 and CDKN1B), further suggesting the interactions observed between the genes of interest.

The interaction between the proteins DLC1 and CDK6 was shown to have a dominant-negative effect, as suggested by the significant risky effect of aA:bb and protective effect of aA:BB of the identified SNP pair. Several related proteins show distinct patterns for tumors having the aA:bb genotype combination. This evidence suggests that the DLC1 heterodimers (gene product of aA) adversely affect the wild-type homodimers (products of AA), resulting in a poorer prognosis than the mutant homodimers (the product of aa). In other words, DLC1 expression is the lowest in rare homozygous patients (aa), and that of functional DLC1 is the lowest in patients harboring aA:bb among all the genotype combinations.

In this study, we have linked the germline genetic polymorphisms and their synergistic effect with breast cancer progression, which provides the basis for experimentally elucidating the mechanisms involved in differential progression. The findings are particularly useful in investigating inherited susceptibility and their cooperative roles in tumor progression, with the ultimate goal of tailoring clinical treatments for breast cancer patients based on their genetic susceptibility.

## Supplementary Material

Supporting Information
