# Supplementary material for: Cooperation of DLC1 and CDK6 Affects Breast Cancer Clinical Outcome
Source: G3 (Bethesda). 2014 Nov 24;5(1):81–91. doi: 10.1534/g3.114.014894 (PMC4291472; doi:10.1534/g3.114.014894)
Supplement: Supporting Information [file supp_5_1_81__index.html]

Cooperation of DLC1 and CDK6 Affects Breast Cancer Clinical Outcome — Cooperation of DLC1 and CDK6 Affects Breast Cancer Clinical Outcome — Supporting Information 

# Cooperation of DLC1 and CDK6 Affects Breast Cancer Clinical Outcome

## Supporting Information for Dai *et al.*, 2015

**Files in this Data Supplement:**

- Supporting Information - Figures S1-S6 and Tables S1-S13 (PDF, 722 KB)
- Figure S1 - Kaplan Meier plots on patients' survival showing interactions between the identified SNP pair of DLC1 (rs561681) and CDK6 (rs3731343). (PDF, 280 KB)
- Figure S2 - Kaplan Meier plots on patients' survival showing interactions between DLC1 and CDK6 gene expression. (PDF, 128 KB)
- Figure S3 - Kaplan Meier plots on patients' survival showing interactions between the expression of proteins directly related to DLC1 and CDK6 (caveolin1 binds DLC1, and CDKN1B and CyclinD1 are related to CDK6). (PDF, 159 KB)
- Figure S4 - Kaplan-Meier plots on patient survival using protein expression data of proteins related to DLC1 and CDK6. (PDF, 142 KB)
- Figure S5 - Protein profiles showing significant distinct patterns for tumors harboring the aA:bb genotype as compared with the other genotype combinations. (PDF, 165 KB)
- Figure S6 - Network predicted from IPA. (PDF, 155 KB)
- Table S1 - Data sets description. (PDF, 97 KB)
- Table S2 - SNP pairs showing significant consistent synergy effect using HEBCS and POSH data. (PDF, 98 KB)
- Table S3 - Detailed statistics of the model with the interacting term by fitting the 'overdominant+additive' model. (PDF, 119 KB)
- Table S4 - Copy number variation correlation between DLC1 and genes under interest. (PDF, 95 KB)
- Table S5 - Model selection for fitting the relevant protein data. (PDF, 116 KB)
- Table S6 - Statistics of the selected model including the interactions between proteins closely related to DLC1 and CDK6. (PDF, 96 KB)
- Table S7 - Proteins differentially expressed among groups categorized by the genotype combinations of the identified SNP pair. (PDF, 95 KB)
- Table S8 - Correlations among proteins showing distinct profiles across genotype combination of the SNP pair. (PDF, 117 KB)
- Table S9 - DLC1 profiles with respect to the genotype of rs532841 (DLC1) and genotype combinations with the rs3739298 (CDK6). (PDF, 117 KB)
- Table S10 - Tagging SNPs of rs561681 having potential functional effects on DLC1. (PDF, 95 KB)
- Table S11 - The phenotypic association of some histopathological markers with the genotype of the DLC1 SNP rs561681. (PDF, 100 KB)
- Table S12 - Tagging SNPs of rs561681 and their influences on DLC1. (PDF, 143 KB)
- Table S13 - Correlations between DLC1 and genes under interest. (PDF, 119 KB)
