# Supplementary material for: Cooperation of DLC1 and CDK6 Affects Breast Cancer Clinical Outcome
Source: G3 (Bethesda). 2014 Nov 24;5(1):81–91. doi: 10.1534/g3.114.014894 (PMC4291472; doi:10.1534/g3.114.014894)
Supplement: Supporting Information [file supp_g3.114.014894_014894SI.pdf]

G3 · Genes | Genomes | Genetics

Cooperation of DLC1 and CDK6 affects breast cancer clinical outcome

Xiaofeng Dai[1, 2], Lu Li[1], Xiuxia Liu[1], Weiguo Hu[1], Yankun Yang[1]*, Zhonghu Bai[1]*

1: National Engineering Laboratory for Cereal Fermention Technology, School of Biotechnology, JiangNan University, Wuxi 214122, China

2: Department of Obstetrics and Gynecology, University of Helsinki and Helsinki University Central Hospital, Helsinki, Finland

*Corresponding authors: Yankun Yang, No. 1800 Lihu Avenue, Wuxi, Jiangsu, 214122, China. +86-510-85329306, yangyankun@jiangnan.edu.cn, or Zhonghu Bai, No. 1800 Lihu Avenue, Wuxi, Jiangsu, 214122, China. +86-510-85329306, baizhonghu@jiangnan.edu.cn (Both contributed equally to this work).

DOI: 10.1534/g3.114.014894

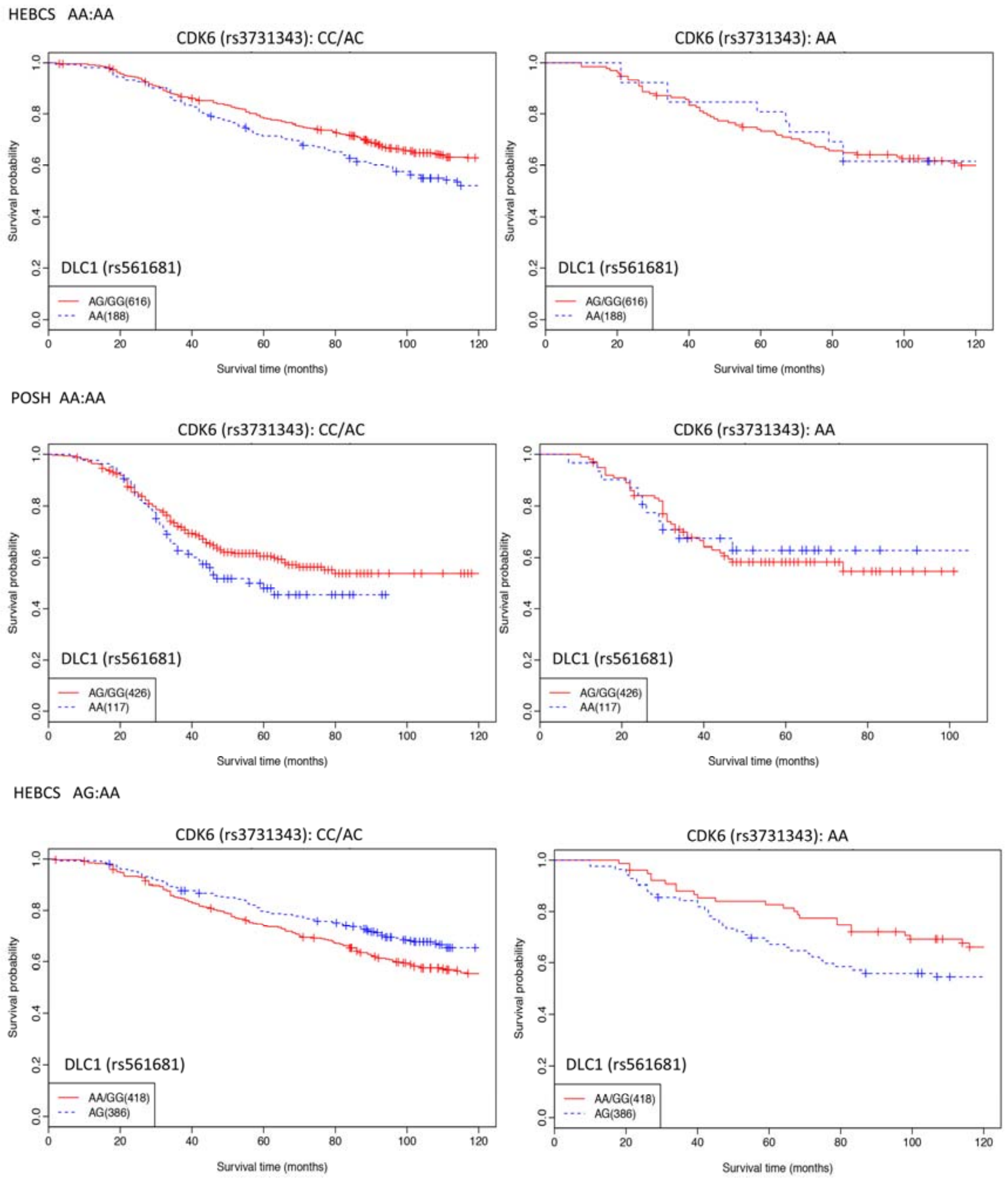

Figure S1 Kaplan Meier plots on patients' survival showing interactions between the identified SNP pair of DLC1 (rs561681) and CDK6 (rs3731343). The first 4 panels (AA:AA) show the interaction between the rare homozygotes of the SNP pair, the intermediate 4 panels (AG:AA) show the interaction between the heterozygote of the DLC1 SNP and the rare homozygote of the CDK6 SNP, and the last 4 panels (AG:CC) show the interaction between the heterozygote of the DLC1 SNP and the common homozygote of the CDK6 SNP.

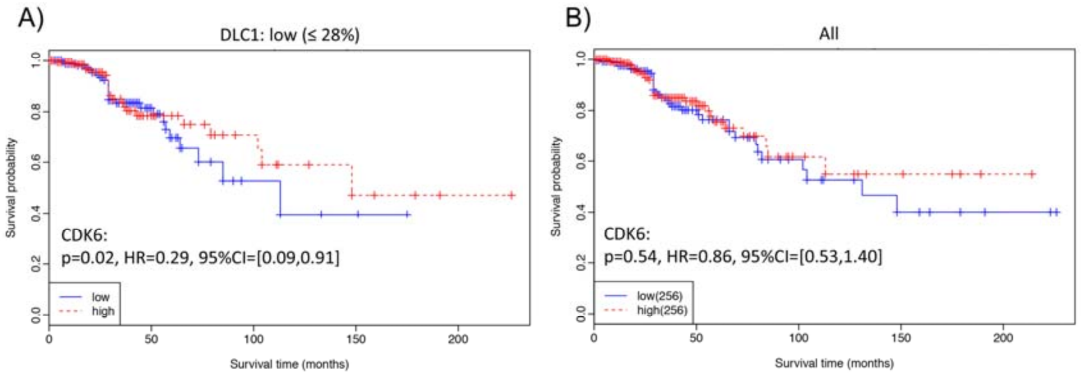

Figure S2 Kaplan Meier plots on patients' survival showing interactions between DLC1 and CDK6 gene expression. A) Kaplan Meier plots on patient survival for DLC1 gene expression when the expression level of CDK6 and DLC1 are below 74% and 28% percentile of all the samples, respectively. B) Kaplan Meier plots on patient survival for DLC1 gene expression (median was used to split the gene expression of CDK6 into high and low expression) when all samples are included.

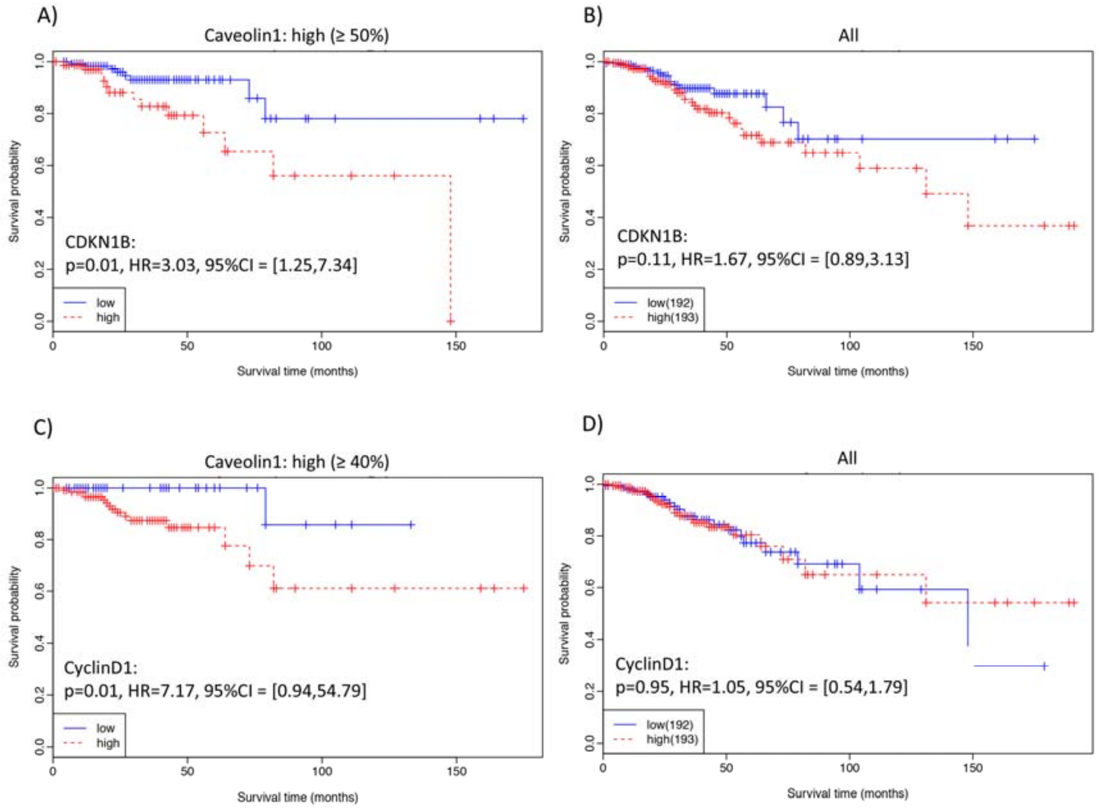

Figure S3 Kaplan Meier plots on patients' survival showing interactions between the expression of proteins directly related to DLC1 and CDK6 (caveolin1 binds DLC1, and CDKN1B and CyclinD1 are related to CDK6). A) Kaplan Meier plot for the protein expression of CDKN1B when caveolin 1 is highly expressed (≥50% expression level). B) Kaplan Meier plots for the protein expression of CDKN1B when all samples are included C) Kaplan Meier plots for the protein expression of cyclin D1 when caveolin 1 is highly expressed (≥50% expression level). D) Kaplan Meier plots for the protein expression of CDKN1B when all samples are included. In all the subplots, median was used to split the expression of CDKN1B or cyclin D1 into high and low expression.

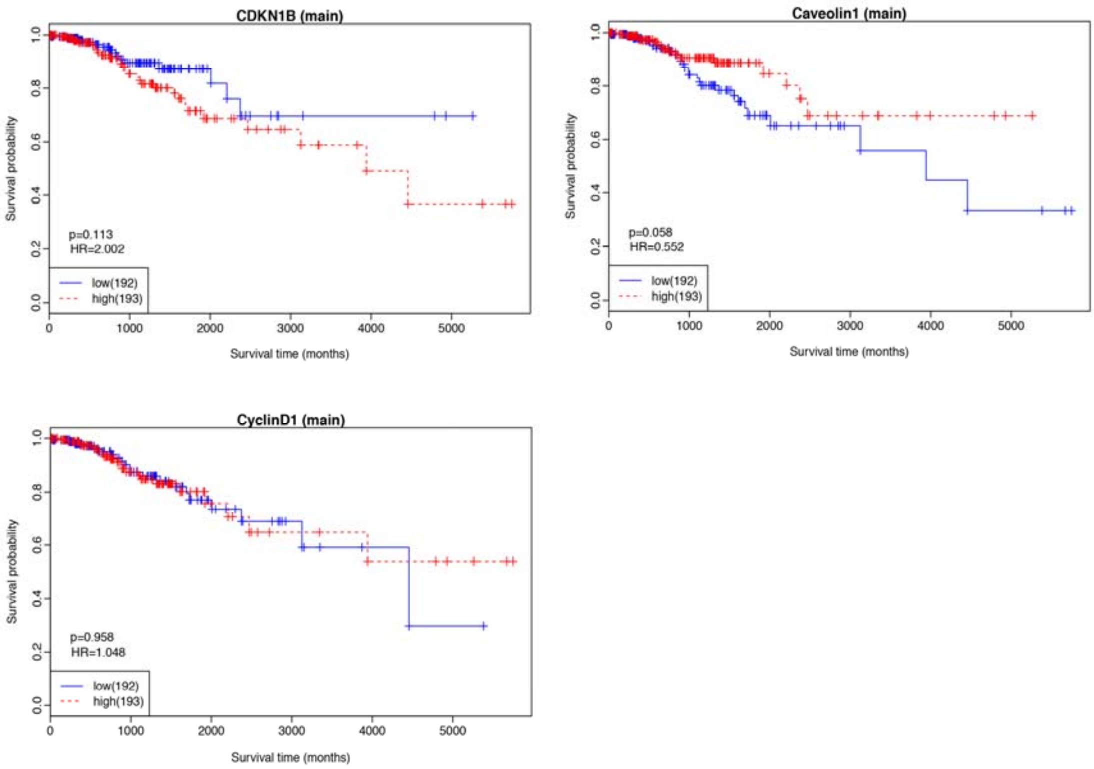

Figure S4 Kaplan-Meier plots on patient survival using protein expression data of proteins related to DLC1 and CDK6. Caveolin1 binds DLC1, and CDKN1B and CyclinD1 are related to CDK6 (details see the main text).

A) CDKN1B

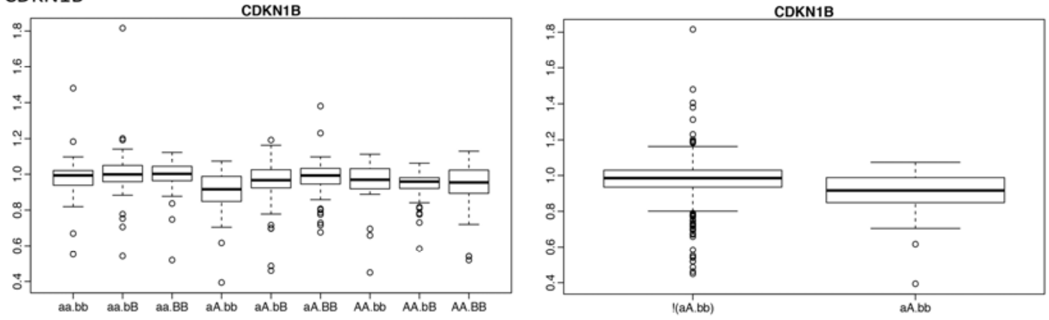

B) PECAM1

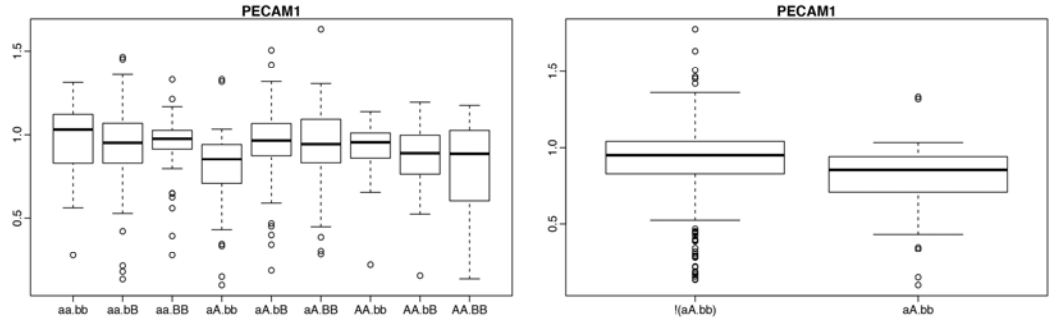

C) p53

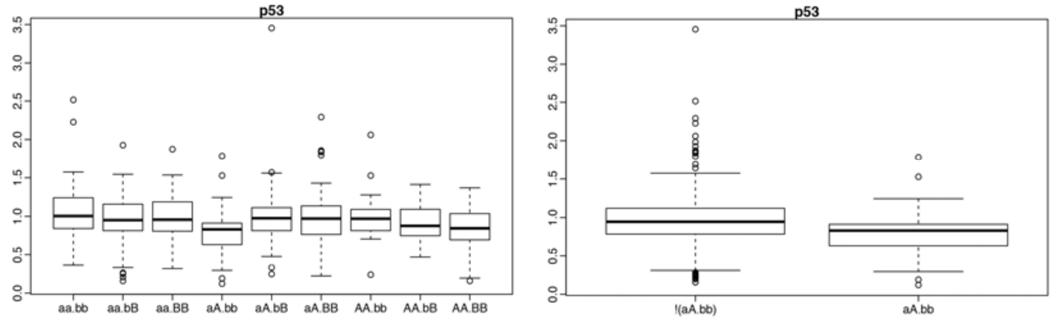

D) Caveolin 1

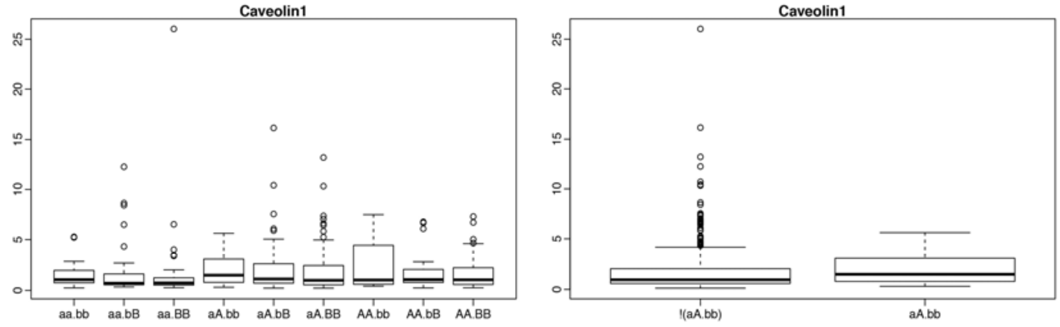

Figure S5 Protein profiles showing significant distinct patterns for tumors harboring the aA:bb genotype as compared with the other genotype combinations. Protein profiles categorized by all the genotype combinations are shown in the left panel of each subplot and those comparing the aA.bb genotype with the

other combinations are shown in the right panel of each subplot. 'aA.bb' means the heterozygote of the DLC1 SNP combined with the rare homozygote of the CDK6 SNP, and !(aA.bb) represents tumors do not harbor this genotype combination.

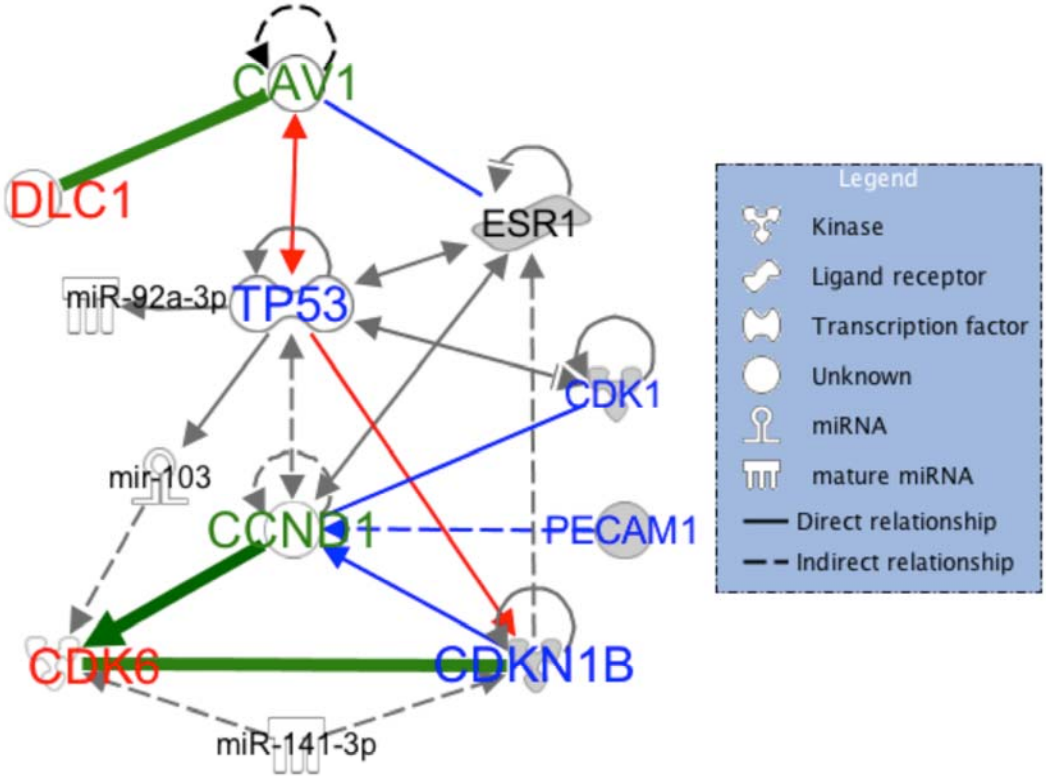

Figure S6 Network predicted from IPA. Only connections with evidence from experiment are included.

Connections without arrow indicate physical binding.

Table S1 Data sets description. 'SNP' stands for the genotype and phenotype data, 'GEX' represents gene expression data, 'PEX' is short for protein expression data, and 'CNV' means copy number variation. The total number of samples is shown for each data set, and the number of events in data used for survival analysis is shown in the bracket.

	SNP			GEX		PEX	CNV
	**HEBCS**	**POSH**	**TCGA**	**HEBCS**	**TCGA**	**TCGA**	**TCGA**
**Total**	**805 (312)**	**543 (220)**	**502**	**183**	**514 (65)**	**385**	**889**
SNP survival	805	543					
GEX survival					514		
GEX correlation				183	514		
PEX correlation						385	
CNV correlation							889
GEX by genotype			502		502		
PEX by genotype			385			385	
CNV by genotype			502				502

Table S2 SNP pairs showing significant consistent synergy effect using HEBCS and POSH data. 'SNP1_DLC1' and 'SNP2_CDK6' are the DLC1 and CDK6 SNPs, respectively. 'Group' shows the samples used in the analysis, i.e., 'main' means using all samples, 'ERn' using ER negative samples, 'ERp' using ER positive samples. 'SNP1_eQTL' and 'SNP2_eQTL' show whether the DLC1 SNP has significant association with DLC1, and the CDK6 SNP has significant association with CDK6, respectively.

SNP1_DLC1	SNP2_CDK6	Group	SNP1_eQTL	SNP2_eQTL
rs609020	rs2237572	main	no	no
rs561681	rs2237572	main	DLC1	no
rs609020	rs3731343	main	no	CDK6
rs561681	rs3731343	main	DLC1	CDK6
rs1372707	rs2282978	main	no	no
rs1372707	rs11765954	main	no	no
rs12541254	rs2282983	main	no	no
rs485673	rs1544342	main	no	no
rs485673	rs10953073	main	no	no
rs485673	rs11762350	main	no	no
rs1372707	rs2282983	main	no	no
rs2410025	rs1544342	main	no	no
rs2410025	rs10953073	main	no	no
rs1372707	rs2282979	main	no	no
rs6531022	rs2237572	ERn	no	no
rs609020	rs2237572	ERn	no	no
rs561681	rs2237572	ERn	DLC1	no
rs532841	rs7781436	ERn	no	no
rs550020	rs7781436	ERn	no	no
rs1372707	rs445	ERn	no	CDK6
rs485673	rs2374589	ERn	no	CDK6
rs2410025	rs2374594	ERn	no	CDK6
rs2410025	rs2374589	ERn	no	CDK6
rs485673	rs2374594	ERn	no	CDK6
rs633979	rs9640606	ERp	no	no
rs633979	rs2079147	ERp	no	no
rs1372707	rs2282978	ERp	no	no
rs1372707	rs2282983	ERp	no	no
rs9325866	rs8	ERp	no	no
rs9325866	rs8179	ERp	no	no

Table S3 Detailed statistics of the model with the interacting term by fitting the 'overdominant+additive' model. 'Genotype' shows the true genotypes and genotype combinations in the pair, where 'G1' and 'G2' means gene 1 (DLC1) and gene 2 (CDK6), respectively. 'Symbol' shows the symbols representing the genotypes where 'a', 'A' are the rare and common allele in the DLC1 SNP and 'b' and 'B' are those for the CDK6 SNP, respectively. 'HR', '95%CI' and 'p' are the hazard ratio, 95% confidence interval ([low,high]) and p value for the analysis.

			**HEBCS**			**POSH**			**POOL**		
**Gene**	**Genotype**	**Symbol**	**HR**	**95%CI**	**p**	**HR**	**95%CI**	**p**	**HR**	**95%CI**	**p**
G1	AG	aA	0.52	[0.34,0.80]	0.0034	0.577	[0.35,0.96]	0.0320	0.55	[0.40,0.76]	3.80E-04
G2	AC	bB	0.89	[0.64,1.24]	0.5100	0.763	[0.50,1.14]	0.1900	0.84	[0.65,1.09]	0.1700
G2	AA	bb	0.65	[0.41,1.04]	0.0730	0.607	[0.37,1.01]	0.0510	0.63	[0.45,0.89]	0.0083
G1:G2	AG:AC	aA:bB	1.65	[0.97,2.82]	0.0660	1.505	[0.80,2.84]	0.2100	1.56	[1.04,2.35]	0.0320
G1:G2	AG:AA	aA:bb	2.97	[1.52,5.79]	0.0014	2.471	[1.18,5.18]	0.0170	2.74	[1.67,4.50]	6.60E-05

Table S4 Copy number variation correlation between DLC1 and genes under interest. Statistics are from the linear model.

Genes	cor	p
CDK6	0.18133	0.0008427
CDK4	-0.07139	0.06075
CDKN2A	0.13183	0.0006614
CDKN2B	0.13235	0.0006267
P53	0.19322	0.0001806
CDKN1B	-0.0307	0.429
CDK1	-0.0548	0.1268
PECAM1	-0.06851	0.002374

Table S5 Model selection for fitting the relevant protein data.

Proteins	Caveolin1:CDKN1B	Caveolin1:CyclinD1
p (M1)	0.0148	0.0106
p (M2)	0.1589	0.1336
p (M1M2)	0.0090	0.0073

M1: G1+G2+G1*G2

M2: G1+G2

Table S6 Statistics of the selected model including the interactions between proteins closely related to DLC1 and CDK6. 'Protein' shows the protein and protein pair, where Caveolin 1 binds DLC1, and CDKN1B and CyclinD1 are related to CDK6 (details see the text). 'Level' shows the expression level, with the expression percentile shown in the bracket. 'HR', '95%CI' and 'p' are the hazard ratio, 95% confidence interval ([low, high]), and p value for each protein or protein pair, respectively.

Protein	Level	HR	95%CI	p	Protein	Level	HR	95%CI	p
CDKN1B	high (≥ 50%)	0.34	[0.21,1.30]	0.1604	CyclinD1	high (≥ 50%)	0.66	[0.28,1.57]	0.3507
Caveolin1	high (≥ 40%)	0.80	[0.08,0.61]	0.00366	Caveolin1	high (≥ 50%)	0.09	[0.01,0.69]	0.0206
CDKN1B:Caveolin1	high:high	5.67	[1.60,20.11]	0.00722	CyclinD1:Caveolin1	high:high	11.06	[1.22,100.01]	0.0325

Table S7 Proteins differentially expressed among groups categorized by the genotype combinations of the identified SNP pair. The aA:bb genotype combination is compared with all the rest combinations. Kruskal-Wallis rank sum test was used. 'Level' shows the relative expression level of DLC1 in aA:bb tumors compared with the others.

**Proteins**	**Genes**	**p**	**Level**
CDKN1B	CDKN1B	0.0014	low
PECAM1	PECAM1	0.0024	low
p53	TP53	0.0024	low
Claudin7	CLDN7	0.0127	low
CHK1	CHEK1	0.0160	low
Transglutaminase2	TGM2	0.0194	low
ACC	ACACA	0.0231	high
PRDX1	PRDX1	0.0274	low
GSK3α&β	GSK3A&B	0.0328	high
PKCα	PRKCA	0.0467	high
Caveolin1	CAV1	0.0764	high

Table S8 Correlations among proteins showing distinct profiles across genotype combination of the SNP pair. Linear model (LM) and correlation test (CorTest) were built between the protein expressions of each protein pair. Values shown in the upper-right triangle are the correlation coefficients of the linear model, and values shown in the lower-left triangle are the corresponding p values.

LM	Caveolin1	CDKN1B	Cyclin D1	PECAM1	P53	CDK1
**Caveolin1**		-0.0194	0.0830	-3.8940	-2.6016	-1.7936
**CDKN1B**	3.09E-12		-0.5054	0.3180	0.1596	0.2011
**Cyclin D1**	2.29E-14	0.0115		0.0492	-0.2469	-0.0353
**PECAM1**	4.33E-16	< 2.2E-16	0.6553		0.9034	0.4037
**P53**	1.98E-15	< 2.2E-16	0.0010	< 2.2E-16		0.6536
**CDK1**	0.0008	6.70E-12	0.7658	< 2.2E-16	< 2.2E-16	
**CorTest**	**Caveolin1**	**CDKN1B**	**Cyclin D1**	**PECAM1**	**P53**	**CDK1**
**Caveolin1**		-0.3455	0.3759	-0.3983	-0.3899	-0.1706
**CDKN1B**	3.09E-12		-0.1287	0.5789	0.4258	0.3404
**Cyclin D1**	2.29E-14	0.0115		0.0228	0.0010	-0.0152
**PECAM1**	4.33E-16	< 2.2E-16	0.6553		0.6165303	0.4342
**P53**	1.98E-15	< 2.2E-16	-0.1677	< 2.2E-16		0.4147
**CDK1**	0.0008	6.70E-12	0.7658	< 2.2E-16	< 2.2E-16	

Table S9 DLC1 profiles with respect to the genotype of rs532841 (DLC1) and genotype combinations with the rs3739298 (CDK6). 'GEX' and 'CNV' represent the gene expression and copy number variation profile, respectively. 'add:add' stands for the genotype combination of the SNP pair after fitting both SNPs to the additive model; 'rec:add' means fitting rs532841 (DLC1) to the recessive model and rs3739298 (CDK6) to the additive model, respectively before genotype combination. 'p_cor' is the p value of the correlation test (cor.test from R) and 'p_kw' is the p value of the Kruskal-Wallis rank sum test (kruskal.test from R).

DLC1	GEX			CNV		
	**p_cor**	**cor**	**p_kw**	**p_cor**	**cor**	**p_kw**
rs561681	0.0040	0.135	0.0233	0.0001	0.189	0.0001
add:add	0.0021	0.153	0.1533	0.0003	0.175	0.0031
rec:add	0.0174	-0.113	0.0653	3.21E-05	-0.198	0.0010

Table S10 Tagging SNPs of rs561681 having potential functional effects on DLC1. ‘Distance’, ‘r2’ and ‘d’ ’ show the distance, r square and d prime of the tagging SNP to rs561681. ‘Region’ shows the SNP location in DLC1. ‘Effect’ shows the influence of the rare allele of the SNP (details in text). ‘Risk’ shows the ranking of the SNP risk from FASTSNP, i.e., 1: very low; 2: low; 3: medium.

SNP	Distance	r2	d'	Region	Effect	Risk
rs532841	6450	0.652	0.955	Coding	Missense: V791M	2~3
rs3739298	7000	0.517	1	Coding	Synonymous: 607, 170	2~3
rs621554	7334	0.817	1	Intron	TFBS loss: AP-1, CREB	1~2

Table S11 The phenotypic association of some histopathological markers with the genotype of the DLC1 SNP rs561681.

rs561681 (DLC1)		AA		aA		aa		
	Groups	count	%	count	%	count	%	Group categorization details
Cyclin D1	0	28	19.40%	71	29.20%	15	13.50%	Negative
	1	116	80.60%	172	70.80%	96	86.50%	Positive
	p_chisq	0.002						
	p_linear	0.415418537						
Cyclin E	0	77	53.50%	153	63.00%	59	53.20%	Negative
	1	67	46.50%	90	37.00%	52	46.80%	Positive
	p_chisq	0.093						
	p_linear	0.88353773						
ER	0	54	26.5%	117	32.3%	57	33.1%	Negative
	1	150	73.5%	245	67.7%	115	66.9%	Positive
	p	0.270						
	p_trend	0.150						
PR	0	84	41.2%	155	42.9%	76	44.4%	Negative
	1	120	58.8%	206	57.1%	95	55.6%	Positive
	p_chisq	0.814						
	p_linear	0.522						
HER2	0	122	84.1%	189	81.1%	91	82.7%	Negative
	1	23	15.9%	44	18.9%	19	17.3%	Positive
	p_chisq	0.750						
	p_linear	0.721						
P53	0	107	77.0%	178	75.4%	80	76.9%	Negative
	1	32	23.0%	58	24.6%	24	23.1%	Positive

	p_chisq	0.925						
	p_linear	0.960						
**Ki67**	0	37	19.2%	59	18.0%	32	20.9%	Negative
	1	73	37.8%	129	39.3%	54	35.3%	Weak positive (5-19%)
	2	36	18.7%	72	22.0%	26	17.0%	Moderate positive (20-30%)
	3	47	24.4%	68	20.7%	41	26.8%	Strong positive (>30%)
	p_chisq	0.643						
	p_linear	0.923						
**Histopathological Type**	1	165	71.7%	269	70.1%	136	73.9%	Ductal carcinoma
	2	38	16.5%	78	20.3%	34	18.5%	Lobular carcinoma
	3	5	2.2%	4	1.0%	3	1.6%	Medullary carcinoma
	4	22	9.6%	33	8.6%	11	6.0%	Other
	p_chisq	0.635						
	p_linear	0.274						
**Tumour Grade**	1	39	18.4%	70	19.9%	34	20.2%	Low grade
	2	97	45.8%	146	41.5%	68	40.5%	Intermediate grade
	3	76	35.8%	136	38.6%	66	39.3%	High grade
	p_chisq	0.853						
	p_linear	0.828						
**Tumour Size Status**	1	119	52.7%	192	50.8%	79	43.2%	Tumour <= 2 cm in greatest dimension
	2	78	34.5%	143	37.8%	80	43.7%	Tumour >2 cm but not >5 cm in greatest dimension
	3	17	7.5%	24	6.3%	10	5.5%	Tumour >5 cm in greatest dimension
	4	12	5.3%	19	5.0%	14	7.7%	Tumour of any size with direct extension to chest wall or skin or both

	p_chisq	0.360						
	p_linear	0.162						
**Regional Lymph Nodes Metastasis**	0	94	41.8%	161	42.9%	79	44.1%	No regional lymph node metastasis
	1	131	58.2%	214	57.1%	100	55.9%	Lymph node metastasis
	p_chisq	0.893						
	p_linear	0.634						
**Distant Metastasis**	0	213	93.4%	352	92.1%	172	94.5%	No distant metastasis
	1	15	6.6%	30	7.9%	10	5.5%	Distant metastasis
	p_chisq	0.569						
	p_linear	0.725						

Table S12 Tagging SNPs of rs561681 and their influences on DLC1.

TaggingSNP	AAchange	TF_remove	TF_add	Distance	r2	D'	Variant	Risk
rs532841	V791M			6450	0.652	0.955	missense(conservative), splicing regulation	2~3
rs11203494	D255N			405793	0.013	1	missense(conservative)	2~3
rs3816748	L81V			406315	0.013	1	missense(conservative), splicing regulation (ESE motif diminished)	2~3
rs3739298	607, 170			7000	0.517	1	synonymous, splicing regulation	2~3
rs568182	1182, 745			710	0.055	1	synonymous, splicing regulation	2~3
rs658856	1167, 730			1268	0.055	1	synonymous, splicing regulation	2~3
rs621554		AP-1 (M00173)		7334	0.817	1	intronic enhancer	1~2
		CREB (M00039)						
rs2280335		CRE-BP (M00040)		9256	0.652	0.955	intronic enhancer	1~2
rs13282126			GATA-1 (M00075)	3459	0.715	1	intronic enhancer	1~2
			GATA-2 (M00076)					
rs13249541			STATx (M00223)	17871	0.652	0.955	intronic enhancer	1~2
rs3736512			OCT-x (M00210)	19748	0.652	0.955	intronic enhancer	1~2
rs17514			MZF1 (M00083)					1~2
			STATx (M00223)					
rs3779993			SRY (M00148)	10351	0.517	1	intronic enhancer	1~2
rs674979			SRY (M00148)	98	0.231	1	intronic enhancer	1~2
rs1729128		CREB (M00039)		258128	0.085	1	intronic enhancer	1~2

	CRE-BP						
	(M00040)						
rs7828964		TATA (M00252)	76887	0.07	1	intronic enhancer	1~2
rs1431206		SRY (M00148)	80536	0.07	1	intronic enhancer	1~2
rs1349958		GATA-2 (M00076)	239408	0.07	1	intronic enhancer	1~2
	GATA-1						
rs1671368	(M00075)		239689	0.07	1	intronic enhancer	1~2
	Tst-1 (M00133)						
	GATA-2						
rs6989950	(M00076)		250629	0.069	1	intronic enhancer	1~2
rs1729136		C/EBPb (M00109)	229134	0.062	1	intronic enhancer	1~2
rs1671384		GATA-1 (M00075)	234180	0.062	1	intronic enhancer	1~2
		GATA-2 (M00076)					
rs1454949		SRY (M00148)	236804	0.062	1	intronic enhancer	1~2
rs1671367	USF (M00217)		239853	0.062	1	intronic enhancer	1~2
	Arnt (M00236)						
rs1454945		SRY (M00148)	241236	0.062	1	intronic enhancer	1~2
		c-Rel (M00053)					
	AML-1a						
rs1454939	(M00271)		242773	0.062	1	intronic enhancer	1~2
rs17802731		OCT-1 (M00137)	256718	0.062	1	intronic enhancer	1~2
		Pbx-1 (M00096)					
rs1620120		OCT-1 (M00137)	245623	0.057	1	intronic enhancer	1~2
rs1729120		C/EBP (M00159)	249035	0.057	1	intronic enhancer	1~2
rs7007799		MZF1 (M00083)	144182	0.055	1	intronic enhancer	1~2
rs1624786		SRY (M00160)	226234	0.055	1	intronic enhancer	1~2
	GATA-1						
rs1653035	(M00075)		226531	0.055	1	intronic enhancer	1~2

rs1378255		OCT-1 (M00137)	230157	0.055	1	intronic enhancer	1~2
rs1671388		C/EBP (M00159)	231401	0.055	1	intronic enhancer	1~2
rs1729147	GATA-1 (M00075)	Elk-1 (M00007)	232434	0.055	1	intronic enhancer	1~2
rs1671380		OCT-x (M00210)	235381	0.055	1	intronic enhancer	1~2
rs1729098	AP-1 (M00199)		237552	0.055	1	intronic enhancer	1~2
rs1729099		GATA-2 (M00076)	237647	0.055	1	intronic enhancer	1~2
rs1454948		NF-E2 (M00037)	238325	0.055	1	intronic enhancer	1~2
rs1349961	Pbx-1 (M00096)		239210	0.055	1	intronic enhancer	1~2
rs1349959		GATA-1 (M00075)	239407	0.055	1	intronic enhancer	1~2
rs1671366	GATA-1 (M00075)		239860	0.055	1	intronic enhancer	1~2
rs1671364		GATA-1 (M00075)	239944	0.055	1	intronic enhancer	1~2
rs1671363	E4BP4 (M00045)		240112	0.055	1	intronic enhancer	1~2
rs1729108	OCT-1 (M00137)		243217	0.055	1	intronic enhancer	1~2
rs1729109		OCT-1 (M00137)	243303	0.055	1	intronic enhancer	1~2
rs1671356		HSF2 (M00147)	243600	0.055	1	intronic enhancer	1~2
rs1671355		C/EBPb (M00117)	243920	0.055	1	intronic enhancer	1~2
rs595003		C/EBPa (M00116) GATA-2 (M00076)	2889	0.047	1	intronic enhancer	1~2
rs10091884		SRY (M00148)	234392	0.047	1	intronic enhancer	1~2
rs4831408	GATA-2 (M00076)	Pbx-1 (M00096)	261559	0.047	1	intronic enhancer	1~2
rs17091956	GATA-1 (M00075)		252921	0.045	1	intronic enhancer	1~2

rs17092222		OCT-1 (M00162)	279382	0.045	1	intronic enhancer	1~2
rs11779801	SRY (M00148)		31030	0.04	1	intronic enhancer	1~2
rs17128435	AP-1 (M00173)		196632	0.04	1	intronic enhancer	1~2
rs919588		GATA-1 (M00075)	227277	0.04	1	intronic enhancer	1~2
		GATA-2 (M00076)					
rs17093957		GATA-1 (M00075)	392315	0.04	1	intronic enhancer	1~2
	GATA-1						
rs7826507	(M00075)	Pbx-1 (M00096)	201187	0.034	1	intronic enhancer	1~2
rs1454952	SRY (M00160)		228344	0.034	1	intronic enhancer	1~2
	AML-1a						
rs1454935	(M00271)		247412	0.034	1	intronic enhancer	1~2
rs1729116		HSF2 (M00147)	247767	0.034	1	intronic enhancer	1~2
rs1729117		HSF2 (M00147)	248001	0.034	1	intronic enhancer	1~2
rs1671346		HSF2 (M00147)	248396	0.034	1	intronic enhancer	1~2
rs11787237	IRF-1 (M00062)	SRY (M00148)	152937	0.033	1	intronic enhancer	1~2
rs17793289		Tst-1 (M00133)	160271	0.033	1	intronic enhancer	1~2
rs7816046	SRY (M00148)		309760	0.033	1	intronic enhancer	1~2
	C/EBP						
	(M00159)						
rs961729		c-Rel (M00053)	379772	0.033	1	intronic enhancer	1~2
	GATA-2						
rs1628187	(M00076)		225849	0.026	1	intronic enhancer	1~2
	GATA-3						
	(M00077)						
rs1671354		OCT-1 (M00137)	244310	0.026	1	intronic enhancer	1~2
rs1454938	YY1 (M00059)		244525	0.026	1	intronic enhancer	1~2
rs7839254		Elk-1 (M00007)	263271	0.026	1	intronic enhancer	1~2
rs172322		TATA (M00252)	294959	0.026	1	intronic enhancer	1~2

	C/EBP						
rs992858	(M00159)		369480	0.026	1	intronic enhancer	1~2
	OCT-1						
rs1871815	(M00137)		417115	0.026	1	intronic enhancer	1~2
rs7005118		OCT-1 (M00162)	417733	0.026	1	intronic enhancer	1~2
		USF (M00217)					
rs17094404	AP-1 (M00173)		419118	0.026	1	intronic enhancer	1~2
rs10503449		USF (M00122)	247831	0.022	1	intronic enhancer	1~2
rs1729119		SRY (M00148)	249007	0.022	1	intronic enhancer	1~2
rs6530628		C/EBP (M00159)	254602	0.022	1	intronic enhancer	1~2
rs9918762		GATA-1 (M00075)	410343	0.022	1	intronic enhancer	1~2
rs11994421		GATA-1 (M00075)	143160	0.02	1	intronic enhancer	1~2
		GATA-2 (M00076)					
	GATA-1						
rs11785676	(M00075)	USF (M00122)	275920	0.02	1	intronic enhancer	1~2
rs17216264	TATA (M00216)		296611	0.02	1	intronic enhancer	1~2
	CDP CR						
	(M00106)						
rs387051		SRY (M00148)	330306	0.02	1	intronic enhancer	1~2
		TATA (M00252)					
rs4831442		CDP CR (M00106)	394389	0.02	1	intronic enhancer	1~2
		Pbx-1 (M00096)					
rs17219640	HSF1 (M00146)		368487	0.013	1	intronic enhancer	1~2
rs3943252		OCT-1 (M00137)	403248	0.013	1	intronic enhancer	1~2
		HNF-1 (M00206)					
rs7820887		AML-1a (M00271)	414591	0.013	1	intronic enhancer	1~2
rs6530639	TATA (M00252)	Pbx-1 (M00096)	418919	0.013	1	intronic enhancer	1~2
	OCT-1						

	(M00162)						
rs13265830	GATA-1 (M00075) GATA-2 (M00076)	MZF1 (M00084)	39263	0.011	1	intronic enhancer	1~2
rs12544071	C/EBPb (M00109)		96991	0.011	1	intronic enhancer	1~2
rs1671336		GATA-1 (M00075)	254148	0.011	1	intronic enhancer	1~2
rs2291210	OCT-1 (M00137)		314527	0.011	1	intronic enhancer	1~2
rs28649074	GATA-1 (M00126) GATA-1 (M00077) GATA-X (M00203)		362067	0.011	1	intronic enhancer	1~2
rs7832786		GATA-1 (M00126) GATA-1 (M00077) GATA-X (M00203)	374749	0.011	1	intronic enhancer	1~2
rs1653021	TATA (M00252)		199781	0.006	1	intronic enhancer	1~2
rs4083255	GATA-1 (M00075) GATA-2 (M00076) GATA-3 (M00077)		222670	0.006	1	intronic enhancer	1~2
rs289560	TATA (M00216)		298324	0.006	1	intronic enhancer	1~2

Table S13 Correlations between DLC1 and genes under interest. 'main', 'ERp', 'ERn' means using all the samples, ER positive samples, ER negative samples in the analysis, respectively. 'p_spearman_HEBCS' and 'cor_spearman_HEBCS' are the p value and correlation obtained from spearman test using HEBCS data. 'p_lm_HEBCS' and 'cor_lm_HEBCS' are the p value and correlation score obtained from linear model using HEBCS data. 'p_spearman_TCGA' and 'cor_spearman_TCGA' are the p value and correlation obtained from spearman test using TCGA data. 'p_lm_TCGA' and 'cor_lm_TCGA' are the p value and correlation score obtained from linear model using TCGA data.

**main**	**p_spearman_HEBCS**	**cor_spearman_HEBCS**	**p_lm_HEBCS**	**cor_lm_HEBCS**	**p_spearman_TCGA**	**cor_spearman_TCGA**	**p_lm_TCGA**	**cor_lm_TCGA**	**Significance**
CDK6	0.312336891	0.075094841	0.312336891	0.052193419	0.081730155	0.076854021	0.081730155	0.05610992	
CDK4	0.010681741	-0.188317676	0.010681741	-0.315493772	1.17E-10	-0.279197913	1.17E-10	-0.454230915	*
CDKN2A	1.63E-06	-0.345808923	1.63E-06	0.00891642	-0.115249379	0.00891642	-0.060785817	*	
CDKN2B	0.828739856	-0.016099972	0.828739856	-0.027471097	9.37E-05	0.171444829	9.37E-05	0.142745914	
**ERp**	**p_spearman_HEBCS**	**cor_spearman_HEBCS**	**p_lm_HEBCS**	**cor_lm_HEBCS**	**p_spearman_TCGA**	**cor_spearman_TCGA**	**p_lm_TCGA**	**cor_lm_TCGA**	**Significance**
CDK6	0.013176245	0.211331288	0.013176245	0.184406073	2.18E-09	0.296725001	2.18E-09	0.276449073	*
CDK4	0.464714579	-0.062977576	0.464714579	-0.107142059	4.50E-07	-0.251896819	4.50E-07	-0.408551012	
CDKN2A	0.00886513	-0.222826572	0.00886513	-0.32502442	0.021991322	0.115817206	0.021991322	0.093232606	
CDKN2B	0.181231751	0.114899326	0.181231751	0.242777043	9.82E-12	0.335400144	9.82E-12	0.33510383	
**ERn**	**p_spearman_HEBCS**	**cor_spearman_HEBCS**	**p_lm_HEBCS**	**cor_lm_HEBCS**	**p_spearman_TCGA**	**cor_spearman_TCGA**	**p_lm_TCGA**	**cor_lm_TCGA**	**Significance**
CDK6	0.66428597	0.066496949	0.66428597	0.041870871	0.464272357	0.069222755	0.464272357	0.04463212	
CDK4	0.005790209	-0.4049622	0.005790209	-0.658088351	0.00180446	-0.289192013	0.00180446	-0.464981075	*
CDKN2A	0.001276336	-0.465373124	0.001276336	-0.374237631	0.013507315	-0.230758368	0.013507315	-0.084179251	*
CDKN2B	0.273199827	-0.166888265	0.273199827	-0.211411132	0.637616727	0.044585754	0.637616727	0.026164307	

* <0.05 for both methods from both populations
