# Supplementary material for: Cooperation of DLC1 and CDK6 Affects Breast Cancer Clinical Outcome
Source: G3 (Bethesda). 2014 Nov 24;5(1):81–91. doi: 10.1534/g3.114.014894 (PMC4291472; doi:10.1534/g3.114.014894)
Supplement: Supporting Information [file supp_g3.114.014894_FigureS1.pdf]

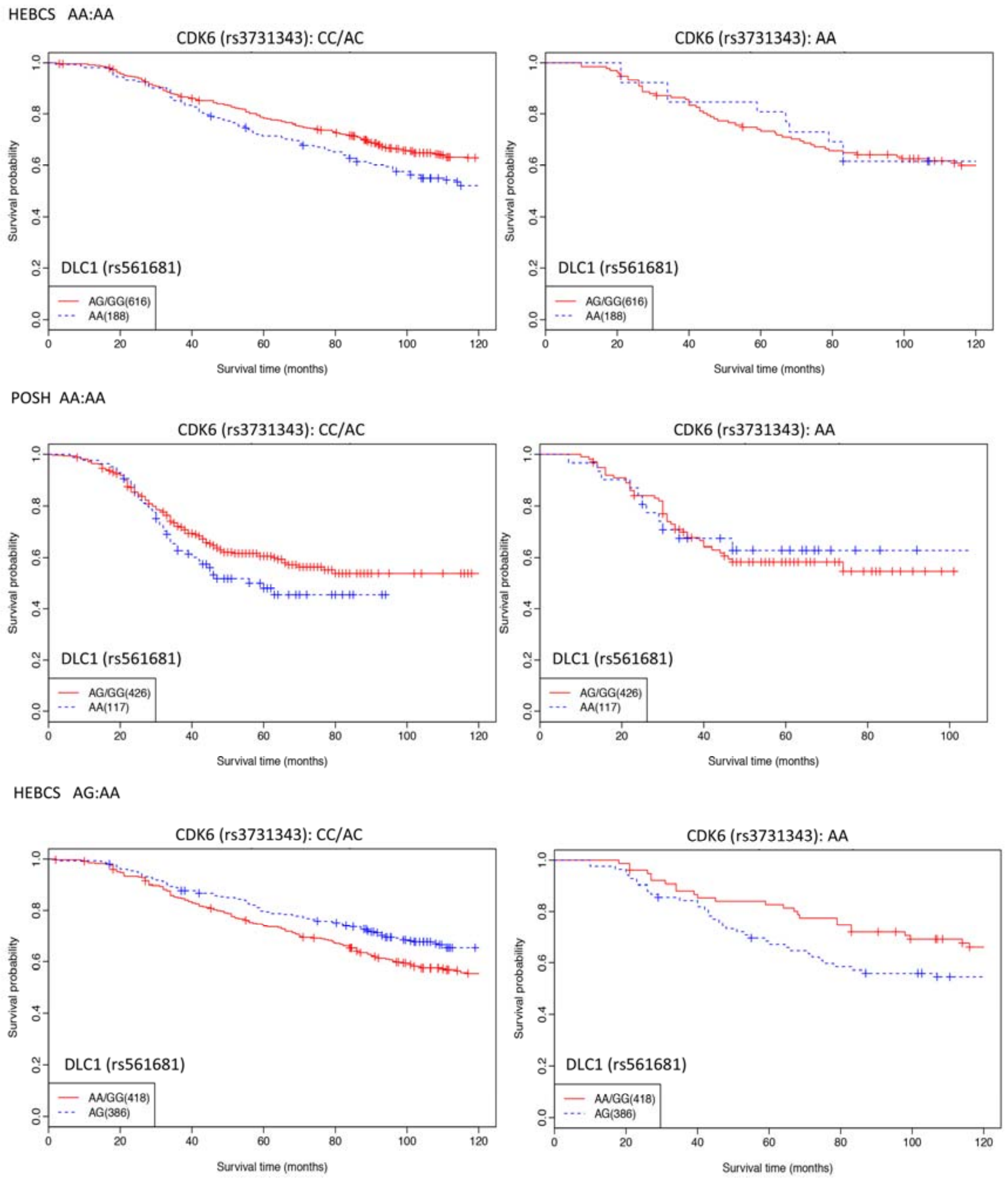


**Figure S1** Kaplan Meier plots on patients' survival showing interactions between the identified SNP pair of DLC1 (rs561681) and CDK6 (rs3731343). The first 4 panels (AA:AA) show the interaction between the rare homozygotes of the SNP pair, the intermediate 4 panels (AG:AA) show the interaction between the heterozygote of the DLC1 SNP and the rare homozygote of the CDK6 SNP, and the last 4 panels (AG:CC) show the interaction between the heterozygote of the DLC1 SNP and the common homozygote of the CDK6 SNP.
