# Supplementary material for: Cooperation of DLC1 and CDK6 Affects Breast Cancer Clinical Outcome
Source: G3 (Bethesda). 2014 Nov 24;5(1):81–91. doi: 10.1534/g3.114.014894 (PMC4291472; doi:10.1534/g3.114.014894)
Supplement: Supporting Information [file supp_g3.114.014894_FigureS2.pdf]

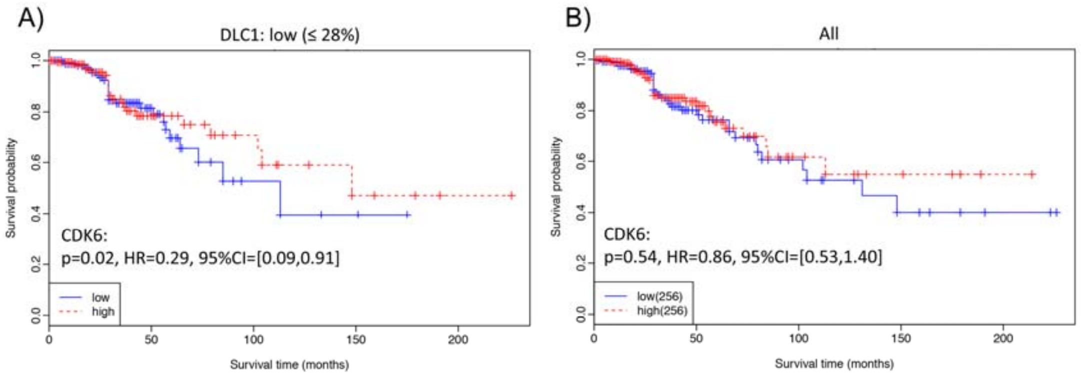

Figure S2 Kaplan Meier plots on patients' survival showing interactions between DLC1 and CDK6 gene expression. A) Kaplan Meier plots on patient survival for DLC1 gene expression when the expression level of CDK6 and DLC1 are below 74% and 28% percentile of all the samples, respectively. B) Kaplan Meier plots on patient survival for DLC1 gene expression (median was used to split the gene expression of CDK6 into high and low expression) when all samples are included.
