# Supplementary material for: Cooperation of DLC1 and CDK6 Affects Breast Cancer Clinical Outcome
Source: G3 (Bethesda). 2014 Nov 24;5(1):81–91. doi: 10.1534/g3.114.014894 (PMC4291472; doi:10.1534/g3.114.014894)
Supplement: Supporting Information [file supp_g3.114.014894_FigureS3.pdf]

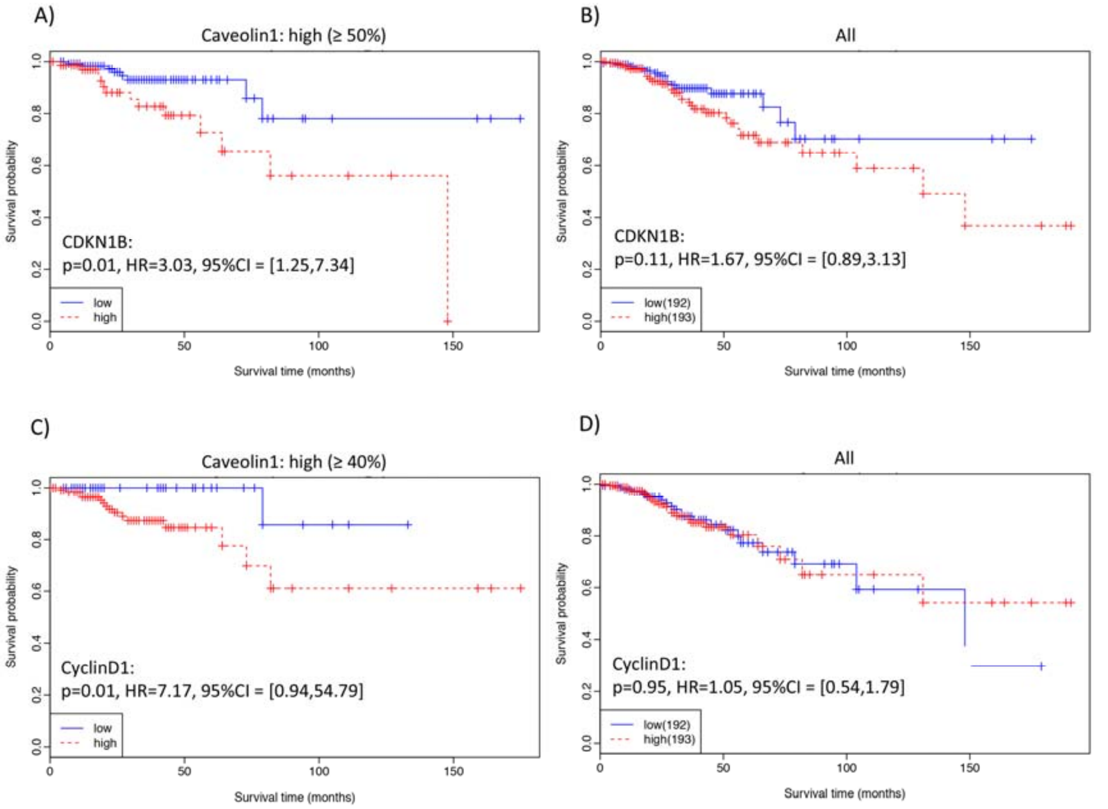

Figure S3 Kaplan Meier plots on patients' survival showing interactions between the expression of proteins directly related to DLC1 and CDK6 (caveolin1 binds DLC1, and CDKN1B and CyclinD1 are related to CDK6). A) Kaplan Meier plot for the protein expression of CDKN1B when caveolin 1 is highly expressed (≥50% expression level). B) Kaplan Meier plots for the protein expression of CDKN1B when all samples are included C) Kaplan Meier plots for the protein expression of cyclin D1 when caveolin 1 is highly expressed (≥50% expression level). D) Kaplan Meier plots for the protein expression of CDKN1B when all samples are included. In all the subplots, median was used to split the expression of CDKN1B or cyclin D1 into high and low expression.
