# Supplementary material for: Cooperation of DLC1 and CDK6 Affects Breast Cancer Clinical Outcome
Source: G3 (Bethesda). 2014 Nov 24;5(1):81–91. doi: 10.1534/g3.114.014894 (PMC4291472; doi:10.1534/g3.114.014894)
Supplement: Supporting Information [file supp_g3.114.014894_FigureS4.pdf]

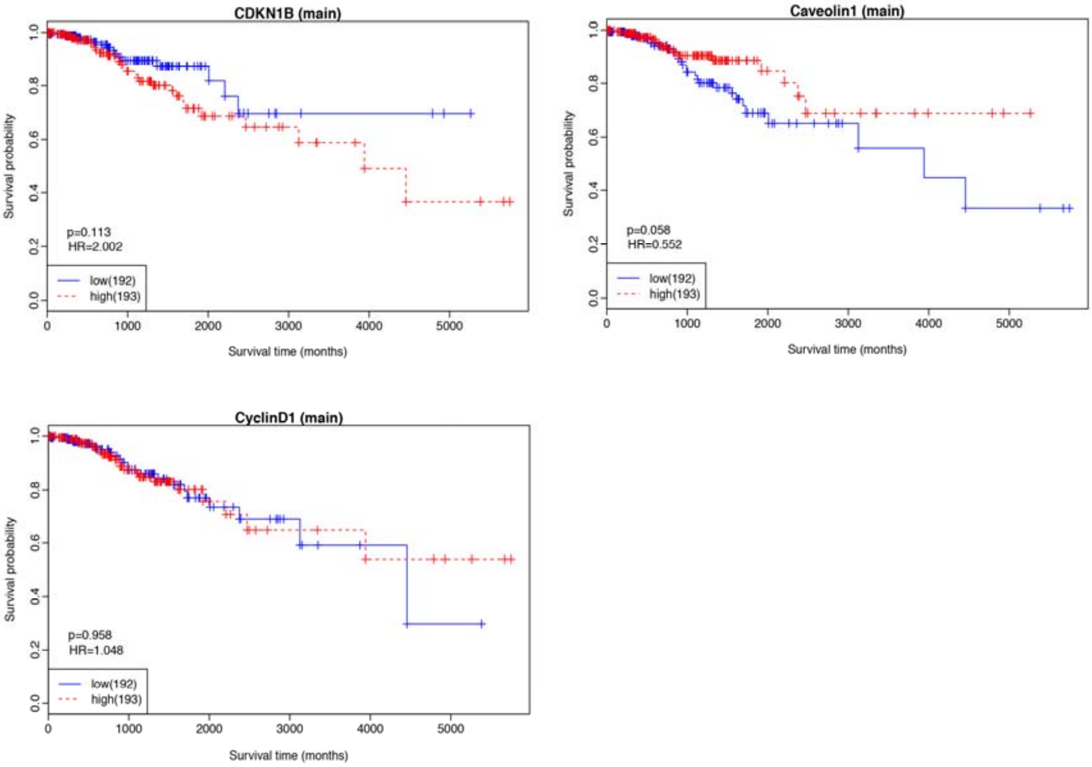

Figure S4 Kaplan-Meier plots on patient survival using protein expression data of proteins related to DLC1 and CDK6. Caveolin1 binds DLC1, and CDKN1B and CyclinD1 are related to CDK6 (details see the main text).
