# Supplementary material for: Cooperation of DLC1 and CDK6 Affects Breast Cancer Clinical Outcome
Source: G3 (Bethesda). 2014 Nov 24;5(1):81–91. doi: 10.1534/g3.114.014894 (PMC4291472; doi:10.1534/g3.114.014894)
Supplement: Supporting Information [file supp_g3.114.014894_FigureS5.pdf]

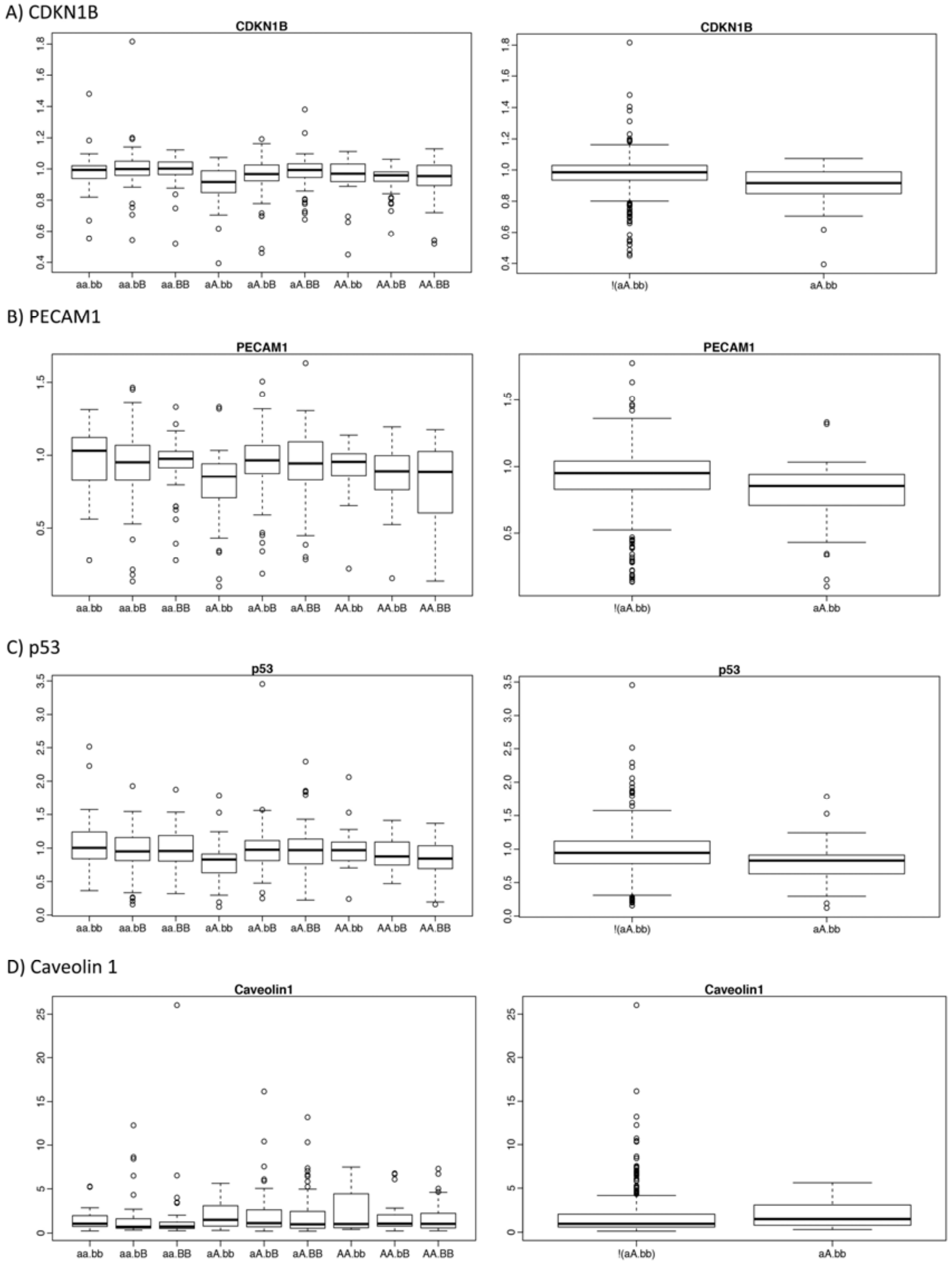

Figure S5 Protein profiles showing significant distinct patterns for tumors harboring the aA:bb genotype as compared with the other genotype combinations. Protein profiles categorized by all the genotype combinations are shown in the left panel of each subplot and those comparing the aA.bb genotype with the

other combinations are shown in the right panel of each subplot. ‘aA.bb’ means the heterozygote of the DLC1 SNP combined with the rare homozygote of the CDK6 SNP, and !(aA.bb) represents tumors do not harbor this genotype combination.
