# Supplementary material for: Cooperation of DLC1 and CDK6 Affects Breast Cancer Clinical Outcome
Source: G3 (Bethesda). 2014 Nov 24;5(1):81–91. doi: 10.1534/g3.114.014894 (PMC4291472; doi:10.1534/g3.114.014894)
Supplement: Supporting Information [file supp_g3.114.014894_TableS1.pdf]

**Table S1** Data sets description. 'SNP' stands for the genotype and phenotype data, 'GEX' represents gene expression data, 'PEX' is short for protein expression data, and 'CNV' means copy number variation. The total number of samples is shown for each data set, and the number of events in data used for survival analysis is shown in the bracket.

| | **SNP** | | | **GEX** | | **PEX** | **CNV** |
|---|---|---|---|---|---|---|---|
| | **HEBCS** | **POSH** | **TCGA** | **HEBCS** | **TCGA** | **TCGA** | **TCGA** |
| **Total** | **805 (312)** | **543 (220)** | **502** | **183** | **514 (65)** | **385** | **889** |
| SNP survival | 805 | 543 | | | | | |
| GEX survival | | | | | 514 | | |
| GEX correlation | | | | 183 | 514 | | |
| PEX correlation | | | | | | 385 | |
| CNV correlation | | | | | | | 889 |
| GEX by genotype | | | 502 | | 502 | | |
| PEX by genotype | | | 385 | | | 385 | |
| CNV by genotype | | | 502 | | | | 502 |
