# Supplementary material for: Cooperation of DLC1 and CDK6 Affects Breast Cancer Clinical Outcome
Source: G3 (Bethesda). 2014 Nov 24;5(1):81–91. doi: 10.1534/g3.114.014894 (PMC4291472; doi:10.1534/g3.114.014894)
Supplement: Supporting Information [file supp_g3.114.014894_TableS2.pdf]

Table S2 SNP pairs showing significant consistent synergy effect using HEBCS and POSH data. 'SNP1_DLC1' and 'SNP2_CDK6' are the DLC1 and CDK6 SNPs, respectively. 'Group' shows the samples used in the analysis, i.e., 'main' means using all samples, 'ERn' using ER negative samples, 'ERp' using ER positive samples. 'SNP1_eQTL' and 'SNP2_eQTL' show whether the DLC1 SNP has significant association with DLC1, and the CDK6 SNP has significant association with CDK6, respectively.

**SNP1_DLC1**	**SNP2_CDK6**	**Group**	**SNP1_eQTL**	**SNP2_eQTL**
rs609020	rs2237572	main	no	no
rs561681	rs2237572	main	DLC1	no
rs609020	rs3731343	main	no	CDK6
rs561681	rs3731343	main	DLC1	CDK6
rs1372707	rs2282978	main	no	no
rs1372707	rs11765954	main	no	no
rs12541254	rs2282983	main	no	no
rs485673	rs1544342	main	no	no
rs485673	rs10953073	main	no	no
rs485673	rs11762350	main	no	no
rs1372707	rs2282983	main	no	no
rs2410025	rs1544342	main	no	no
rs2410025	rs10953073	main	no	no
rs1372707	rs2282979	main	no	no
rs6531022	rs2237572	ERn	no	no
rs609020	rs2237572	ERn	no	no
rs561681	rs2237572	ERn	DLC1	no
rs532841	rs7781436	ERn	no	no
rs550020	rs7781436	ERn	no	no
rs1372707	rs445	ERn	no	CDK6
rs485673	rs2374589	ERn	no	CDK6
rs2410025	rs2374594	ERn	no	CDK6
rs2410025	rs2374589	ERn	no	CDK6
rs485673	rs2374594	ERn	no	CDK6
rs633979	rs9640606	ERp	no	no
rs633979	rs2079147	ERp	no	no
rs1372707	rs2282978	ERp	no	no
rs1372707	rs2282983	ERp	no	no
rs9325866	rs8	ERp	no	no
rs9325866	rs8179	ERp	no	no
