# Supplementary material for: Cooperation of DLC1 and CDK6 Affects Breast Cancer Clinical Outcome
Source: G3 (Bethesda). 2014 Nov 24;5(1):81–91. doi: 10.1534/g3.114.014894 (PMC4291472; doi:10.1534/g3.114.014894)
Supplement: Supporting Information [file supp_g3.114.014894_TableS3.pdf]

Table S3 Detailed statistics of the model with the interacting term by fitting the 'overdominant+additive' model. 'Genotype' shows the true genotypes and genotype combinations in the pair, where 'G1' and 'G2' means gene 1 (DLC1) and gene 2 (CDK6), respectively. 'Symbol' shows the symbols representing the genotypes where 'a', 'A' are the rare and common allele in the DLC1 SNP and 'b' and 'B' are those for the CDK6 SNP, respectively. 'HR', '95%CI' and 'p' are the hazard ratio, 95% confidence interval ([low,high]) and p value for the analysis.

			**HEBCS**			**POSH**			**POOL**		
**Gene**	**Genotype**	**Symbol**	**HR**	**95%CI**	**p**	**HR**	**95%CI**	**p**	**HR**	**95%CI**	**p**
G1	AG	aA	0.52	[0.34,0.80]	0.0034	0.577	[0.35,0.96]	0.0320	0.55	[0.40,0.76]	3.80E-04
G2	AC	bB	0.89	[0.64,1.24]	0.5100	0.763	[0.50,1.14]	0.1900	0.84	[0.65,1.09]	0.1700
G2	AA	bb	0.65	[0.41,1.04]	0.0730	0.607	[0.37,1.01]	0.0510	0.63	[0.45,0.89]	0.0083
G1:G2	AG:AC	aA:bB	1.65	[0.97,2.82]	0.0660	1.505	[0.80,2.84]	0.2100	1.56	[1.04,2.35]	0.0320
G1:G2	AG:AA	aA:bb	2.97	[1.52,5.79]	0.0014	2.471	[1.18,5.18]	0.0170	2.74	[1.67,4.50]	6.60E-05
