# Supplementary material for: Cooperation of DLC1 and CDK6 Affects Breast Cancer Clinical Outcome
Source: G3 (Bethesda). 2014 Nov 24;5(1):81–91. doi: 10.1534/g3.114.014894 (PMC4291472; doi:10.1534/g3.114.014894)
Supplement: Supporting Information [file supp_g3.114.014894_TableS4.pdf]

Table S4 Copy number variation correlation between DLC1 and genes under interest. Statistics are from the linear model.

Genes	cor	p
CDK6	0.18133	0.0008427
CDK4	-0.07139	0.06075
CDKN2A	0.13183	0.0006614
CDKN2B	0.13235	0.0006267
P53	0.19322	0.0001806
CDKN1B	-0.0307	0.429
CDK1	-0.0548	0.1268
PECAM1	-0.06851	0.002374
