# Supplementary material for: Cooperation of DLC1 and CDK6 Affects Breast Cancer Clinical Outcome
Source: G3 (Bethesda). 2014 Nov 24;5(1):81–91. doi: 10.1534/g3.114.014894 (PMC4291472; doi:10.1534/g3.114.014894)
Supplement: Supporting Information [file supp_g3.114.014894_TableS5.pdf]

Table S5 Model selection for fitting the relevant protein data.

Proteins	Caveolin1:CDKN1B	Caveolin1:CyclinD1
p (M1)	0.0148	0.0106
p (M2)	0.1589	0.1336
p (M1M2)	0.0090	0.0073

M1: G1+G2+G1*G2

M2: G1+G2
