# Supplementary material for: Cooperation of DLC1 and CDK6 Affects Breast Cancer Clinical Outcome
Source: G3 (Bethesda). 2014 Nov 24;5(1):81–91. doi: 10.1534/g3.114.014894 (PMC4291472; doi:10.1534/g3.114.014894)
Supplement: Supporting Information [file supp_g3.114.014894_TableS6.pdf]

Table S6 Statistics of the selected model including the interactions between proteins closely related to DLC1 and CDK6. 'Protein' shows the protein and protein pair, where Caveolin 1 binds DLC1, and CDKN1B and CyclinD1 are related to CDK6 (details see the text). 'Level' shows the expression level, with the expression percentile shown in the bracket. 'HR', '95%CI' and 'p' are the hazard ratio, 95% confidence interval ([low, high]), and p value for each protein or protein pair, respectively.

Protein	Level	HR	95%CI	p	Protein	Level	HR	95%CI	p
CDKN1B	high (≥ 50%)	0.34	[0.21,1.30]	0.1604	CyclinD1	high (≥ 50%)	0.66	[0.28,1.57]	0.3507
Caveolin1	high (≥ 40%)	0.80	[0.08,0.61]	0.00366	Caveolin1	high (≥ 50%)	0.09	[0.01,0.69]	0.0206
CDKN1B:Caveolin1	high:high	5.67	[1.60,20.11]	0.00722	CyclinD1:Caveolin1	high:high	11.06	[1.22,100.01]	0.0325
