# Supplementary material for: Cooperation of DLC1 and CDK6 Affects Breast Cancer Clinical Outcome
Source: G3 (Bethesda). 2014 Nov 24;5(1):81–91. doi: 10.1534/g3.114.014894 (PMC4291472; doi:10.1534/g3.114.014894)
Supplement: Supporting Information [file supp_g3.114.014894_TableS7.pdf]

Table S7 Proteins differentially expressed among groups categorized by the genotype combinations of the identified SNP pair. The aA:bb genotype combination is compared with all the rest combinations. Kruskal-Wallis rank sum test was used. 'Level' shows the relative expression level of DLC1 in aA:bb tumors compared with the others.

Proteins	Genes	p	Level
CDKN1B	CDKN1B	0.0014	low
PECAM1	PECAM1	0.0024	low
p53	TP53	0.0024	low
Claudin7	CLDN7	0.0127	low
CHK1	CHEK1	0.0160	low
Transglutaminase2	TGM2	0.0194	low
ACC	ACACA	0.0231	high
PRDX1	PRDX1	0.0274	low
GSK3α&β	GSK3A&B	0.0328	high
PKCα	PRKCA	0.0467	high
Caveolin1	CAV1	0.0764	high
