# Supplementary material for: Cooperation of DLC1 and CDK6 Affects Breast Cancer Clinical Outcome
Source: G3 (Bethesda). 2014 Nov 24;5(1):81–91. doi: 10.1534/g3.114.014894 (PMC4291472; doi:10.1534/g3.114.014894)
Supplement: Supporting Information [file supp_g3.114.014894_TableS8.pdf]

Table S8 Correlations among proteins showing distinct profiles across genotype combination of the SNP pair. Linear model (LM) and correlation test (CorTest) were built between the protein expressions of each protein pair. Values shown in the upper-right triangle are the correlation coefficients of the linear model, and values shown in the lower-left triangle are the corresponding p values.

LM	Caveolin1	CDKN1B	Cyclin D1	PECAM1	P53	CDK1
**Caveolin1**		-0.0194	0.0830	-3.8940	-2.6016	-1.7936
**CDKN1B**	3.09E-12		-0.5054	0.3180	0.1596	0.2011
**Cyclin D1**	2.29E-14	0.0115		0.0492	-0.2469	-0.0353
**PECAM1**	4.33E-16	< 2.2E-16	0.6553		0.9034	0.4037
**P53**	1.98E-15	< 2.2E-16	0.0010	< 2.2E-16		0.6536
**CDK1**	0.0008	6.70E-12	0.7658	< 2.2E-16	< 2.2E-16	
**CorTest**	**Caveolin1**	**CDKN1B**	**Cyclin D1**	**PECAM1**	**P53**	**CDK1**
**Caveolin1**		-0.3455	0.3759	-0.3983	-0.3899	-0.1706
**CDKN1B**	3.09E-12		-0.1287	0.5789	0.4258	0.3404
**Cyclin D1**	2.29E-14	0.0115		0.0228	0.0010	-0.0152
**PECAM1**	4.33E-16	< 2.2E-16	0.6553		0.6165303	0.4342
**P53**	1.98E-15	< 2.2E-16	-0.1677	< 2.2E-16		0.4147
**CDK1**	0.0008	6.70E-12	0.7658	< 2.2E-16	< 2.2E-16	
