# Supplementary material for: Cooperation of DLC1 and CDK6 Affects Breast Cancer Clinical Outcome
Source: G3 (Bethesda). 2014 Nov 24;5(1):81–91. doi: 10.1534/g3.114.014894 (PMC4291472; doi:10.1534/g3.114.014894)
Supplement: Supporting Information [file supp_g3.114.014894_TableS9.pdf]

Table S9 DLC1 profiles with respect to the genotype of rs532841 (DLC1) and genotype combinations with the rs3739298 (CDK6). 'GEX' and 'CNV' represent the gene expression and copy number variation profile, respectively. 'add:add' stands for the genotype combination of the SNP pair after fitting both SNPs to the additive model; 'rec:add' means fitting rs532841 (DLC1) to the recessive model and rs3739298 (CDK6) to the additive model, respectively before genotype combination. 'p_cor' is the p value of the correlation test (cor.test from R) and 'p_kw' is the p value of the Kruskal-Wallis rank sum test (kruskal.test from R).

**DLC1**	**GEX**			**CNV**		
	**p_cor**	**cor**	**p_kw**	**p_cor**	**cor**	**p_kw**
rs561681	0.0040	0.135	0.0233	0.0001	0.189	0.0001
add:add	0.0021	0.153	0.1533	0.0003	0.175	0.0031
rec:add	0.0174	-0.113	0.0653	3.21E-05	-0.198	0.0010
