# Supplementary material for: Cooperation of DLC1 and CDK6 Affects Breast Cancer Clinical Outcome
Source: G3 (Bethesda). 2014 Nov 24;5(1):81–91. doi: 10.1534/g3.114.014894 (PMC4291472; doi:10.1534/g3.114.014894)
Supplement: Supporting Information [file supp_g3.114.014894_TableS10.pdf]

Table S10 Tagging SNPs of rs561681 having potential functional effects on DLC1. 'Distance', 'r2' and 'd' ' show the distance, r square and d prime of the tagging SNP to rs561681. 'Region' shows the SNP location in DLC1. 'Effect' shows the influence of the rare allele of the SNP (details in text). 'Risk' shows the ranking of the SNP risk from FASTSNP, i.e., 1: very low; 2: low; 3: medium.

SNP	Distance	r2	d'	Region	Effect	Risk
rs532841	6450	0.652	0.955	Coding	Missense: V791M	2~3
rs3739298	7000	0.517	1	Coding	Synonymous: 607, 170	2~3
rs621554	7334	0.817	1	Intron	TFBS loss: AP-1, CREB	1~2
