# Supplementary material for: Cooperation of DLC1 and CDK6 Affects Breast Cancer Clinical Outcome
Source: G3 (Bethesda). 2014 Nov 24;5(1):81–91. doi: 10.1534/g3.114.014894 (PMC4291472; doi:10.1534/g3.114.014894)
Supplement: Supporting Information [file supp_g3.114.014894_TableS11.pdf]

Table S11 The phenotypic association of some histopathological markers with the genotype of the DLC1 SNP rs561681.

rs561681 (DLC1)		AA		aA		aa		
	Groups	count	%	count	%	count	%	Group categorization details
Cyclin D1	0	28	19.40%	71	29.20%	15	13.50%	Negative
	1	116	80.60%	172	70.80%	96	86.50%	Positive
	p_chisq	0.002						
	p_linear	0.415418537						
Cyclin E	0	77	53.50%	153	63.00%	59	53.20%	Negative
	1	67	46.50%	90	37.00%	52	46.80%	Positive
	p_chisq	0.093						
	p_linear	0.88353773						
ER	0	54	26.5%	117	32.3%	57	33.1%	Negative
	1	150	73.5%	245	67.7%	115	66.9%	Positive
	p	0.270						
	p_trend	0.150						
PR	0	84	41.2%	155	42.9%	76	44.4%	Negative
	1	120	58.8%	206	57.1%	95	55.6%	Positive
	p_chisq	0.814						
	p_linear	0.522						
HER2	0	122	84.1%	189	81.1%	91	82.7%	Negative
	1	23	15.9%	44	18.9%	19	17.3%	Positive
	p_chisq	0.750						
	p_linear	0.721						
P53	0	107	77.0%	178	75.4%	80	76.9%	Negative
	1	32	23.0%	58	24.6%	24	23.1%	Positive

	p_chisq	0.925						
	p_linear	0.960						
**Ki67**	0	37	19.2%	59	18.0%	32	20.9%	Negative
	1	73	37.8%	129	39.3%	54	35.3%	Weak positive (5-19%)
	2	36	18.7%	72	22.0%	26	17.0%	Moderate positive (20-30%)
	3	47	24.4%	68	20.7%	41	26.8%	Strong positive (>30%)
	p_chisq	0.643						
	p_linear	0.923						
**Histopathological Type**	1	165	71.7%	269	70.1%	136	73.9%	Ductal carcinoma
	2	38	16.5%	78	20.3%	34	18.5%	Lobular carcinoma
	3	5	2.2%	4	1.0%	3	1.6%	Medullary carcinoma
	4	22	9.6%	33	8.6%	11	6.0%	Other
	p_chisq	0.635						
	p_linear	0.274						
**Tumour Grade**	1	39	18.4%	70	19.9%	34	20.2%	Low grade
	2	97	45.8%	146	41.5%	68	40.5%	Intermediate grade
	3	76	35.8%	136	38.6%	66	39.3%	High grade
	p_chisq	0.853						
	p_linear	0.828						
**Tumour Size Status**	1	119	52.7%	192	50.8%	79	43.2%	Tumour <= 2 cm in greatest dimension
	2	78	34.5%	143	37.8%	80	43.7%	Tumour >2 cm but not >5 cm in greatest dimension
	3	17	7.5%	24	6.3%	10	5.5%	Tumour >5 cm in greatest dimension
	4	12	5.3%	19	5.0%	14	7.7%	Tumour of any size with direct extension to chest wall or skin or both

	p_chisq	0.360						
	p_linear	0.162						
**Regional Lymph Nodes Metastasis**	0	94	41.8%	161	42.9%	79	44.1%	No regional lymph node metastasis
	1	131	58.2%	214	57.1%	100	55.9%	Lymph node metastasis
	p_chisq	0.893						
	p_linear	0.634						
**Distant Metastasis**	0	213	93.4%	352	92.1%	172	94.5%	No distant metastasis
	1	15	6.6%	30	7.9%	10	5.5%	Distant metastasis
	p_chisq	0.569						
	p_linear	0.725						
