# Supplementary material for: Cooperation of DLC1 and CDK6 Affects Breast Cancer Clinical Outcome
Source: G3 (Bethesda). 2014 Nov 24;5(1):81–91. doi: 10.1534/g3.114.014894 (PMC4291472; doi:10.1534/g3.114.014894)
Supplement: Supporting Information [file supp_g3.114.014894_TableS12.pdf]

Table S12 Tagging SNPs of rs561681 and their influences on DLC1.

TaggingSNP	AAchange	TF_remove	TF_add	Distance	r2	D'	Variant	Risk
rs532841	V791M			6450	0.652	0.955	missense(conservative), splicing regulation	2~3
rs11203494	D255N			405793	0.013	1	missense(conservative)	2~3
rs3816748	L81V			406315	0.013	1	missense(conservative), splicing regulation (ESE motif diminished)	2~3
rs3739298	607, 170			7000	0.517	1	synonymous, splicing regulation	2~3
rs568182	1182, 745			710	0.055	1	synonymous, splicing regulation	2~3
rs658856	1167, 730			1268	0.055	1	synonymous, splicing regulation	2~3
rs621554		AP-1 (M00173)		7334	0.817	1	intronic enhancer	1~2
		CREB (M00039)						
rs2280335		CRE-BP (M00040)		9256	0.652	0.955	intronic enhancer	1~2
rs13282126			GATA-1 (M00075)	3459	0.715	1	intronic enhancer	1~2
			GATA-2 (M00076)					
rs13249541			STATx (M00223)	17871	0.652	0.955	intronic enhancer	1~2
rs3736512			OCT-x (M00210)	19748	0.652	0.955	intronic enhancer	1~2
rs17514			MZF1 (M00083)					1~2
			STATx (M00223)					
rs3779993			SRY (M00148)	10351	0.517	1	intronic enhancer	1~2
rs674979			SRY (M00148)	98	0.231	1	intronic enhancer	1~2
rs1729128		CREB (M00039)		258128	0.085	1	intronic enhancer	1~2

	CRE-BP						
	(M00040)						
rs7828964		TATA (M00252)	76887	0.07	1	intronic enhancer	1~2
rs1431206		SRY (M00148)	80536	0.07	1	intronic enhancer	1~2
rs1349958		GATA-2 (M00076)	239408	0.07	1	intronic enhancer	1~2
	GATA-1						
rs1671368	(M00075)		239689	0.07	1	intronic enhancer	1~2
	Tst-1 (M00133)						
	GATA-2						
rs6989950	(M00076)		250629	0.069	1	intronic enhancer	1~2
rs1729136		C/EBPb (M00109)	229134	0.062	1	intronic enhancer	1~2
rs1671384		GATA-1 (M00075)	234180	0.062	1	intronic enhancer	1~2
		GATA-2 (M00076)					
rs1454949		SRY (M00148)	236804	0.062	1	intronic enhancer	1~2
rs1671367	USF (M00217)		239853	0.062	1	intronic enhancer	1~2
	Arnt (M00236)						
rs1454945		SRY (M00148)	241236	0.062	1	intronic enhancer	1~2
		c-Rel (M00053)					
	AML-1a						
rs1454939	(M00271)		242773	0.062	1	intronic enhancer	1~2
rs17802731		OCT-1 (M00137)	256718	0.062	1	intronic enhancer	1~2
		Pbx-1 (M00096)					
rs1620120		OCT-1 (M00137)	245623	0.057	1	intronic enhancer	1~2
rs1729120		C/EBP (M00159)	249035	0.057	1	intronic enhancer	1~2
rs7007799		MZF1 (M00083)	144182	0.055	1	intronic enhancer	1~2
rs1624786		SRY (M00160)	226234	0.055	1	intronic enhancer	1~2
	GATA-1						
rs1653035	(M00075)		226531	0.055	1	intronic enhancer	1~2

rs1378255		OCT-1 (M00137)	230157	0.055	1	intronic enhancer	1~2
rs1671388		C/EBP (M00159)	231401	0.055	1	intronic enhancer	1~2
rs1729147	GATA-1 (M00075)	Elk-1 (M00007)	232434	0.055	1	intronic enhancer	1~2
rs1671380		OCT-x (M00210)	235381	0.055	1	intronic enhancer	1~2
rs1729098	AP-1 (M00199)		237552	0.055	1	intronic enhancer	1~2
rs1729099		GATA-2 (M00076)	237647	0.055	1	intronic enhancer	1~2
rs1454948		NF-E2 (M00037)	238325	0.055	1	intronic enhancer	1~2
rs1349961	Pbx-1 (M00096)		239210	0.055	1	intronic enhancer	1~2
rs1349959		GATA-1 (M00075)	239407	0.055	1	intronic enhancer	1~2
rs1671366	GATA-1 (M00075)		239860	0.055	1	intronic enhancer	1~2
rs1671364		GATA-1 (M00075)	239944	0.055	1	intronic enhancer	1~2
rs1671363	E4BP4 (M00045)		240112	0.055	1	intronic enhancer	1~2
rs1729108	OCT-1 (M00137)		243217	0.055	1	intronic enhancer	1~2
rs1729109		OCT-1 (M00137)	243303	0.055	1	intronic enhancer	1~2
rs1671356		HSF2 (M00147)	243600	0.055	1	intronic enhancer	1~2
rs1671355		C/EBPb (M00117)	243920	0.055	1	intronic enhancer	1~2
rs595003		C/EBPa (M00116) GATA-2 (M00076)	2889	0.047	1	intronic enhancer	1~2
rs10091884		SRY (M00148)	234392	0.047	1	intronic enhancer	1~2
rs4831408	GATA-2 (M00076)	Pbx-1 (M00096)	261559	0.047	1	intronic enhancer	1~2
rs17091956	GATA-1 (M00075)		252921	0.045	1	intronic enhancer	1~2

rs17092222		OCT-1 (M00162)	279382	0.045	1	intronic enhancer	1~2
rs11779801	SRY (M00148)		31030	0.04	1	intronic enhancer	1~2
rs17128435	AP-1 (M00173)		196632	0.04	1	intronic enhancer	1~2
rs919588		GATA-1 (M00075)<br>GATA-2 (M00076)	227277	0.04	1	intronic enhancer	1~2
rs17093957		GATA-1 (M00075)	392315	0.04	1	intronic enhancer	1~2
rs7826507	GATA-1 (M00075)	Pbx-1 (M00096)	201187	0.034	1	intronic enhancer	1~2
rs1454952	SRY (M00160)		228344	0.034	1	intronic enhancer	1~2
rs1454935	AML-1a (M00271)		247412	0.034	1	intronic enhancer	1~2
rs1729116		HSF2 (M00147)	247767	0.034	1	intronic enhancer	1~2
rs1729117		HSF2 (M00147)	248001	0.034	1	intronic enhancer	1~2
rs1671346		HSF2 (M00147)	248396	0.034	1	intronic enhancer	1~2
rs11787237	IRF-1 (M00062)	SRY (M00148)	152937	0.033	1	intronic enhancer	1~2
rs17793289		Tst-1 (M00133)	160271	0.033	1	intronic enhancer	1~2
rs7816046	SRY (M00148)		309760	0.033	1	intronic enhancer	1~2
rs961729	C/EBP (M00159)	c-Rel (M00053)	379772	0.033	1	intronic enhancer	1~2
rs1628187	GATA-2 (M00076)<br>GATA-3 (M00077)		225849	0.026	1	intronic enhancer	1~2
rs1671354		OCT-1 (M00137)	244310	0.026	1	intronic enhancer	1~2
rs1454938	YY1 (M00059)		244525	0.026	1	intronic enhancer	1~2
rs7839254		Elk-1 (M00007)	263271	0.026	1	intronic enhancer	1~2
rs172322		TATA (M00252)	294959	0.026	1	intronic enhancer	1~2

	C/EBP						
rs992858	(M00159)		369480	0.026	1	intronic enhancer	1~2
	OCT-1						
rs1871815	(M00137)		417115	0.026	1	intronic enhancer	1~2
rs7005118		OCT-1 (M00162)	417733	0.026	1	intronic enhancer	1~2
		USF (M00217)					
rs17094404	AP-1 (M00173)		419118	0.026	1	intronic enhancer	1~2
rs10503449		USF (M00122)	247831	0.022	1	intronic enhancer	1~2
rs1729119		SRY (M00148)	249007	0.022	1	intronic enhancer	1~2
rs6530628		C/EBP (M00159)	254602	0.022	1	intronic enhancer	1~2
rs9918762		GATA-1 (M00075)	410343	0.022	1	intronic enhancer	1~2
rs11994421		GATA-1 (M00075)	143160	0.02	1	intronic enhancer	1~2
		GATA-2 (M00076)					
	GATA-1						
rs11785676	(M00075)	USF (M00122)	275920	0.02	1	intronic enhancer	1~2
rs17216264	TATA (M00216)		296611	0.02	1	intronic enhancer	1~2
	CDP CR						
	(M00106)						
rs387051		SRY (M00148)	330306	0.02	1	intronic enhancer	1~2
		TATA (M00252)					
rs4831442		CDP CR (M00106)	394389	0.02	1	intronic enhancer	1~2
		Pbx-1 (M00096)					
rs17219640	HSF1 (M00146)		368487	0.013	1	intronic enhancer	1~2
rs3943252		OCT-1 (M00137)	403248	0.013	1	intronic enhancer	1~2
		HNF-1 (M00206)					
rs7820887		AML-1a (M00271)	414591	0.013	1	intronic enhancer	1~2
rs6530639	TATA (M00252)	Pbx-1 (M00096)	418919	0.013	1	intronic enhancer	1~2
	OCT-1						

	(M00162)						
rs13265830	GATA-1 (M00075) GATA-2 (M00076)	MZF1 (M00084)	39263	0.011	1	intronic enhancer	1~2
rs12544071	C/EBPb (M00109)		96991	0.011	1	intronic enhancer	1~2
rs1671336		GATA-1 (M00075)	254148	0.011	1	intronic enhancer	1~2
rs2291210	OCT-1 (M00137)		314527	0.011	1	intronic enhancer	1~2
rs28649074	GATA-1 (M00126) GATA-1 (M00077) GATA-X (M00203)		362067	0.011	1	intronic enhancer	1~2
rs7832786		GATA-1 (M00126) GATA-1 (M00077) GATA-X (M00203)	374749	0.011	1	intronic enhancer	1~2
rs1653021	TATA (M00252)		199781	0.006	1	intronic enhancer	1~2
rs4083255	GATA-1 (M00075) GATA-2 (M00076) GATA-3 (M00077)		222670	0.006	1	intronic enhancer	1~2
rs289560	TATA (M00216)		298324	0.006	1	intronic enhancer	1~2
