# Supplementary material for: Cooperation of DLC1 and CDK6 Affects Breast Cancer Clinical Outcome
Source: G3 (Bethesda). 2014 Nov 24;5(1):81–91. doi: 10.1534/g3.114.014894 (PMC4291472; doi:10.1534/g3.114.014894)
Supplement: Supporting Information [file supp_g3.114.014894_TableS13.pdf]

Table S13 Correlations between DLC1 and genes under interest. 'main', 'ERp', 'ERn' means using all the samples, ER positive samples, ER negative samples in the analysis, respectively. 'p_spearman_HEBCS' and 'cor_spearman_HEBCS' are the p value and correlation obtained from spearman test using HEBCS data. 'p_lm_HEBCS' and 'cor_lm_HEBCS' are the p value and correlation score obtained from linear model using HEBCS data. 'p_spearman_TCGA' and 'cor_spearman_TCGA' are the p value and correlation obtained from spearman test using TCGA data. 'p_lm_TCGA' and 'cor_lm_TCGA' are the p value and correlation score obtained from linear model using TCGA data.

**main**	**p_spearman_HEBCS**	**cor_spearman_HEBCS**	**p_lm_HEBCS**	**cor_lm_HEBCS**	**p_spearman_TCGA**	**cor_spearman_TCGA**	**p_lm_TCGA**	**cor_lm_TCGA**	**Significance**
CDK6	0.312336891	0.075094841	0.312336891	0.052193419	0.081730155	0.076854021	0.081730155	0.05610992	
CDK4	0.010681741	-0.188317676	0.010681741	-0.315493772	1.17E-10	-0.279197913	1.17E-10	-0.454230915	*
CDKN2A	1.63E-06	-0.345808923	1.63E-06	0.00891642	-0.115249379	0.00891642	-0.060785817	*	
CDKN2B	0.828739856	-0.016099972	0.828739856	-0.027471097	9.37E-05	0.171444829	9.37E-05	0.142745914	
**ERp**	**p_spearman_HEBCS**	**cor_spearman_HEBCS**	**p_lm_HEBCS**	**cor_lm_HEBCS**	**p_spearman_TCGA**	**cor_spearman_TCGA**	**p_lm_TCGA**	**cor_lm_TCGA**	**Significance**
CDK6	0.013176245	0.211331288	0.013176245	0.184406073	2.18E-09	0.296725001	2.18E-09	0.276449073	*
CDK4	0.464714579	-0.062977576	0.464714579	-0.107142059	4.50E-07	-0.251896819	4.50E-07	-0.408551012	
CDKN2A	0.00886513	-0.222826572	0.00886513	-0.32502442	0.021991322	0.115817206	0.021991322	0.093232606	
CDKN2B	0.181231751	0.114899326	0.181231751	0.242777043	9.82E-12	0.335400144	9.82E-12	0.33510383	
**ERn**	**p_spearman_HEBCS**	**cor_spearman_HEBCS**	**p_lm_HEBCS**	**cor_lm_HEBCS**	**p_spearman_TCGA**	**cor_spearman_TCGA**	**p_lm_TCGA**	**cor_lm_TCGA**	**Significance**
CDK6	0.66428597	0.066496949	0.66428597	0.041870871	0.464272357	0.069222755	0.464272357	0.04463212	
CDK4	0.005790209	-0.4049622	0.005790209	-0.658088351	0.00180446	-0.289192013	0.00180446	-0.464981075	*
CDKN2A	0.001276336	-0.465373124	0.001276336	-0.374237631	0.013507315	-0.230758368	0.013507315	-0.084179251	*
CDKN2B	0.273199827	-0.166888265	0.273199827	-0.211411132	0.637616727	0.044585754	0.637616727	0.026164307	

* <0.05 for both methods from both populations
